# Dissecting two contrasting phytoplankton-symbiont interaction modes based on population dynamics and gene expression patterns

**DOI:** 10.1128/msystems.00803-25

**Published:** 2025-11-04

**Authors:** Jinny Wu Yang, Vincent J. Denef

**Affiliations:** 1 Ecology and Evolutionary Biology, University of Michigan 118714, Ann Arbor, Michigan, USA; 2 Plant Resilience Institute, Michigan State University 3078, East Lansing, Michigan, USA; UiT Norges arktiske universitet Arctic Centre for Sustainable Energy, Tromsø, Norway

**Keywords:** phytoplankton-bacteria interactions, RNA sequencing

## Abstract

Microbial symbionts play vital roles in the health, fitness, and ecological dynamics of most eukaryotic species, making it essential to understand how host-microbe interactions shape the microbiome. Building on our previous work, we hypothesized that symbionts with diverse functions are maintained in the microbiome via a trade-off between two host-microbe interaction modes: either by better utilizing host-derived dissolved organic matter (DOM) without direct interaction with the host (unidirectional interaction) or by engaging in feedback interactions with the host that alter DOM composition to their advantage (bidirectional interaction). By screening symbionts isolated from *C. sorokiniana* (host), we examined growth and gene expression responses of two representative symbionts and the host. We found *Curvibacter* sp. thrived on spent medium from axenic *C. sorokiniana* with host-derived dissolved organic matter (DOM) in unidirectional interaction, whereas *Falsiroseomonas* sp. grew best with live *C. sorokiniana* cells in bidirectional interaction and exhibited a greater shift in gene expression between modes despite larger growth phase differences between treatments for *Curvibacter* sp. Specifically, *Falsiroseomonas* sp. showed differential expression of metabolic pathways that could benefit (e.g., synthesis of cofactors) or antagonize (e.g., metabolism of defensive secondary metabolites) toward the host under bidirectional interaction conditions. In response, host co-cultured with *Falsiroseomonas* sp. reduced its growth and triggered its higher expression of nitrogen-rich amino acid metabolism which may provide a nutritional benefit to *Falsiroseomonas* sp. These findings demonstrated that distinct host-microbe interaction modes drive differential symbiont strategies and play an important role in microbiome assembly.

**IMPORTANCE:**

Deciphering how host-microbe interactions shape microbiome structure is crucial for understanding host health and ecosystem function. Given the inherent complexity of host-microbe interactions, we simplified the system by separating interactions into unidirectional and bidirectional modes. Using this framework, we observed contrasting effects on the growth of two representative bacterial taxa isolated from the same host microbiome. These growth responses were further coupled with distinctive gene expression profiles in both hosts and bacteria under the different interaction modes. Together, these findings underscore the importance of considering host-microbe interaction modes in microbiome research. For example, our findings help explain how hosts can harbor functionally diverse microbial assemblages, where contrasting metabolic strategies are maintained through distinct interaction modes. Such insights are fundamental for predicting, managing, or engineering microbiomes, as well as understanding the ecological processes that drive microbiome diversity and function within host-microbiome systems in nature.

## INTRODUCTION

Most eukaryotic species host a microbiome that is essential to the survival and health of the host organism (1–4). The process of microbiome assembly shapes the composition and diversity of the microbiome and so the functions it provides to the host (5–10). It is well-established that host-microbe interaction is one of the major driving forces in microbiome assembly; however, the intricate mechanisms governing this process remain poorly understood. The composition of the microbiome is often related to host needs, where the host selects beneficial microbes to optimize its fitness, and this selection shifts when the host is under stress or transitions to a different growth phase or life stage (11–13). Several studies have observed dynamic feedback interactions between the host and specific members in the microbiome (14–16), leading to the microbiome itself impacting how the host shapes microbiome composition. Our own recent study showed that the different selection impacts from innate host control versus host-microbiome feedback act together to maintain microbiome composition and diversity (17). This diversity, in turn, may ensure the stability and resilience of microbiome functional contributions to the host (18). Dissecting the effects of host selection and host-microbe feedback on microbiome assembly is a next step in deepening our understanding of the mechanisms that sustain microbiome diversity and functionality.

The phytoplankton-microbiome system serves as an ideal system to dissect the different impacts of host and host-microbe feedback on microbiome assembly. Phytoplankton shape their microbiome through producing dissolved organic matter (DOM) and house their microbes on or within the diffusive boundary layer around cells (12, 19). Such features allow us to combine and separate host, microbiome, and host-produced DOM, enabling us to distinguish the impacts of host selection and host-microbiome feedback on microbiome assembly. While not a universal model for all host-microbiome systems, it provides a tractable model for host microbiome systems where host secretions are the key factor in community assembly. In the wild, the composition of phytoplankton microbiomes is often found correlated to phytoplankton species/genotype and physiological status (e.g., growth phase, under a changing environment, under stress and competition fitness) (8, 20, 21). This suggests ubiquitous and strong phytoplankton-microbiome relationships in the natural system. In laboratory experiments, microbiome composition can be altered and even predicted based on added phytoplankton metabolites (19, 22–25), emphasizing the central role of phytoplankton DOM in shaping microbiome composition. Finally, exposing axenic phytoplankton to bacteria or to different bacterial communities can change phytoplankton gene expression and DOM composition (12, 16, 26, 27) suggesting microbiome members can modify host produced DOM.

In our previous study, we used *Chlorella sorokiniana* as a host model and showed how innate host selection and host-microbiome feedback selected for different microbiome compositions, and when applied together sustained the highest microbiome diversity (17). This finding inspired a hypothesis that different symbionts were selected by different host-microbe interaction modes: (i) innate host selection, a unidirectional interaction mode where the host selects for symbionts which compete best for the DOM innately produced by the host but those symbionts have limited impacts on host physiology; (ii) host-microbe feedback interaction, a bidirectional interaction mode that favors symbionts that have limited ability to compete for DOM innately produced by the host, but directly interact with the host and change its physiology to their benefit. These symbionts may synthesize signaling molecules or metabolites, for example, indole-3-acetic acid, tryptophan, and quorum sensing signals (14, 28), upon encountering the host, thereby modifying host metabolism. Symbiont-produced chemicals may trigger the host to produce metabolites that selectively favor or hinder specific microbial members; for instance, rosmarinic acid has been shown to be produced by the host in response to bacterial co-culture, promoting the growth of beneficial bacteria while inhibiting opportunistic bacteria (12).

To test this hypothesis, we screened isolates obtained from a C. *sorokiniana* microbiome recruited from a natural pond by examining the physiological response (growth) of each isolate when constraining the host-microbe interaction mode to have either only innate host selection available (by introducing host-free DOM in absence of a microbiome) or allowing for the host-microbe feedback interaction to emerge (by introducing host cells after removing the pool of previously produced DOM). We then chose one representative bacterial isolate favoring either host microbe interactions mode and repeated these experiments and explored metabolic (gene expression profiles) responses of the host and each symbiont: the *Curvibacter* sp. that was more favored by unidirectional innate host selection and the *Falsiroseomonas* sp. that was more favored by bidirectional host-microbe feedback interactions. Here, we define symbionts as members that persist in the microbiome and interact with the host, either by being attracted by and consuming host-produced DOM or through direct interaction with the host. Therefore, since both *Curvibacter* sp. and *Falsiroseomonas* sp. are favored by either host-microbe interaction mode, we consider them both symbionts of *C. sorokiniana,* rather than a transient organism that was found in the microbiome by chance.

**Fig 1:**
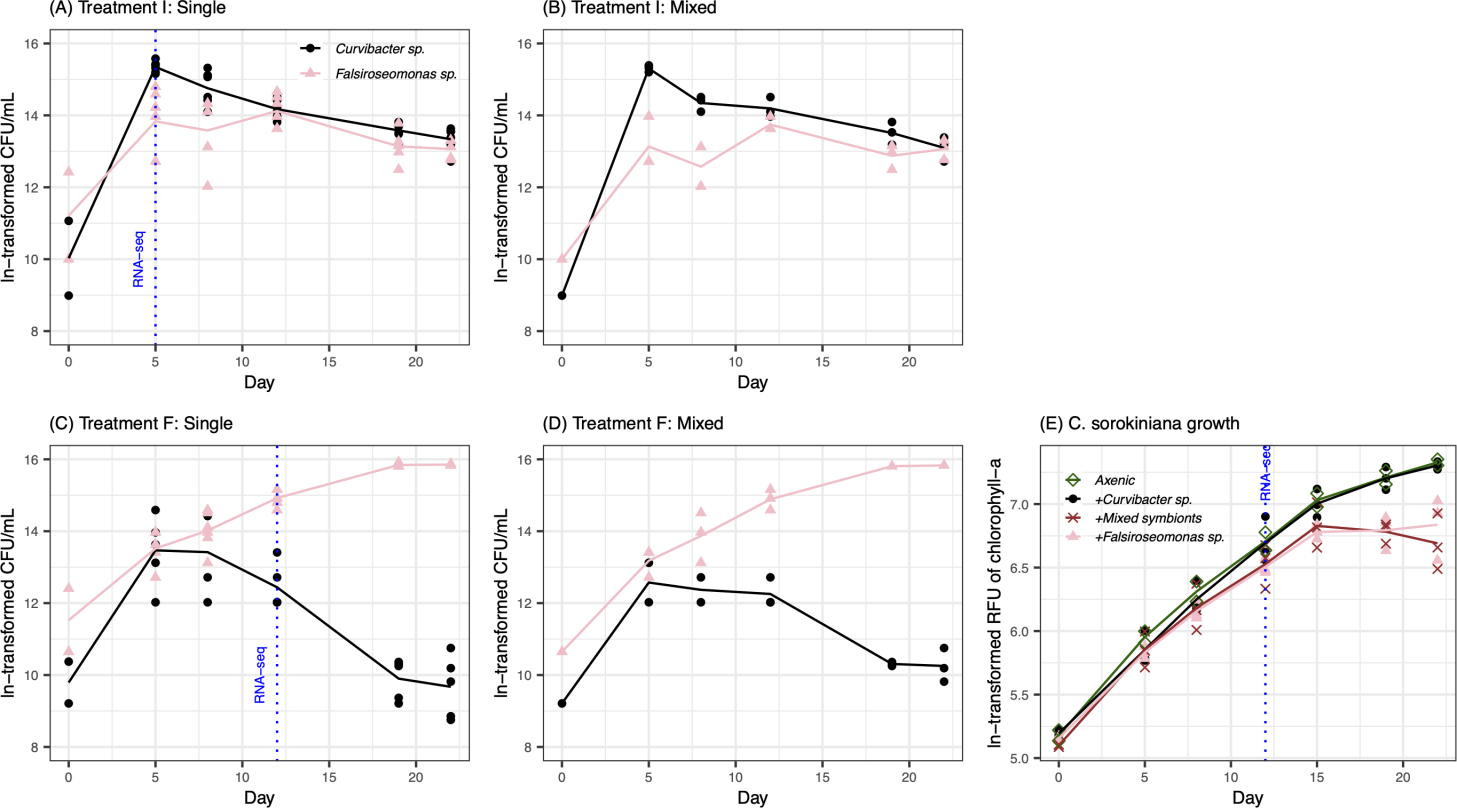
Bacterial and *C. sorokiniana* growth dynamics. Bacterial (**A**) monoculture and (**B**) mixed culture (two bacterial symbionts) in Treatment I (Innate: DOM only) and (**C**) monoculture and (**D**) mixed culture in Treatment F (Feedback: live algal cells), and (**E**) *C. sorokiniana* growth when co-cultured with either bacterium, both bacteria (Mixed), and without bacteria (Axenic). The vertical blue dashed lines indicate the sampling time for RNA sequencing. The bacteria were counted by plate counts (colony-forming unit, CFU) and microalgal growth was measured based on Chlorophyll-*a* fluorescence (RFU). Growth curve lines indicate the mean of replicates, and each point represents a replicate. All measurement values are shown as natural log-transformed (ln-transformed).

Based on their contrasting physiological responses to host-microbe interaction modes, we hypothesized that *Falsiroseomonas* sp. can synthesize signaling molecules or can generate metabolites that trigger feedback interactions with the host that promote *Falsiroseomonas* sp. growth (Fig. 1A and B). In contrast, *Curvibacter* sp. grew better on host DOM than *Falsiroseomonas* sp. but the more limited growth in presence of its host made us hypothesize that it lacks the ability to engage in bidirectional interactions with the host to enhance its growth (Fig. 1C and D). Based on the assumption that synthesizing metabolites and signals requires the expression of different genes to engage in bidirectional interaction, we expected that (i) the *Falsiroseomonas* sp. would exhibit a more significant shift in gene expression between two interaction modes compared to the *Curvibacter* sp. Additionally, (ii) for host C. *sorokiniana* gene expression, we expected a more substantial shift from the axenic state when co-cultured with the *Falsiroseomonas* sp. than with the *Curvibacter* sp. We identified the function of differentially expressed genes with a focus on the function of transporters of host-produced DOM in the symbionts and metabolic pathways in both symbionts and the host. This approach aimed to better understand the extent of gene expression reprogramming and the prioritized metabolic pathways in both the symbionts and the host under different host-microbe interaction modes.

## MATERIALS AND METHODS

### Bacterial and microalgal growth dynamics experiments

The *C. sorokiniana* (UTEX 2805) culture was originally supplied from the University of Texas Culture Collection of Algae in 2011 and rendered axenic in 2018 (29). The axenic culture was maintained on COMBO media slants (30) under a light intensity no higher than 30 µmol · m^−2^· s^−1^ at 15°C and transferred to fresh medium every four to six months since then. Microbiomes were recruited to this axenic culture from natural ponds in 2018 (31). We obtained bacterial isolates in 2021 from those initial microbiomes, and the same microbiomes were also used in our previous experiments to identify the innate and feedback selective effects on microbiome composition (17). We selected six bacteria isolated from xenic *C. sorokiniana* on R2A solid media (Teknova, Hollister, CA, USA) based on their distinctive colony morphology (to enable easy tracking of growth of each culture when in co-culture) and tested their growth response under unidirectional Innate host selection (Treatment I) or bidirectional host-microbiome Feedback (Treatment F). To create two treatments, axenic *C. sorokiniana* was harvested during exponential growth phase (~2 × 10^5^ cells/mL) in COMBO medium, and centrifuged at 900 × *g* for 5 min. The axenicity check was conducted by placing a 10 µL subsample of culture onto a slide, air-drying, fluorescent staining with DAPI, and examining under an epifluorescence microscope to check for microbial contamination. The supernatant was collected and filtered through 1.2 µm pore-size filters to remove any remaining algal cells. This filtered spent medium was used for Treatment I, which contains only dissolved organic matter (DOM) innately produced by the host without any direct microbe-host interactions. For creating Treatment F, the pelleted *C. sorokiniana* cells were washed in fresh COMBO medium to remove remaining spent medium twice and resuspended in fresh COMBO medium at ~2 × 10^5^ cells/mL. This medium contains only axenic host cells without an initial DOM pool. To minimize the impact of remnants of the liquid R2A medium, which the bacterial isolates were grown in, on the microalgal host, each 1 mL bacterial R2A culture was washed twice using COMBO medium by centrifuged at 2,500 × *g* for 10 min, spent medium was removed, and the pellet was resuspended in 1 mL fresh COMBO medium. The final resuspended bacterial isolates were inoculated into each treatment after 1,000 times dilution into five 1 mL replicate cultures using 48-well plates (Fisher Scientific, Hampton, NH, USA). The plates were cultured under a continuous 80 RPM rotation with a 16:8 light/dark cycle under a light intensity of ~80 µmol · m^−2^· s^−1^ at 20°C in a Minitron incubator (Infors HT, Bottmingen, Switzerland). This light intensity and temperature are higher than those used for long-term storage mentioned above to promote higher algal growth and activity. To track bacterial growth, 10 µL of each replicate was sampled for at least five times over a 10- or 12-days period. Colony-forming units (CFU) were counted using the drop plate technique (Herigstad, Hamilton, and Heersink 2001), where each 10 µL subsample was 10^−2^, 10^−3^, and 10^−4^ diluted using R2A liquid medium, and three 10 µL replicate drops of each dilution were dropped on R2A agar plates. The plates were incubated at room temperature in the dark for 5 days.

After two representative bacterial symbionts were identified, we repeated the experiment with a larger culture volume that would be needed to obtain sufficient RNA for transcriptome analysis. We grew the *Curvibacter* sp. and *Falsiroseomonas* sp. in Treatment I and Treatment F following the aforementioned procedure in 100 mL volume in 125 mL glass Erlenmeyer flasks in triplicate. In addition to mono-bacterial cultures, we also grew mixed-bacteria cultures in each treatment condition to investigate potential effects on bacterial growth patterns from microbe-microbe interactions. In the larger volume experiments, 10 µL of each culture was collected on day 0, 5, 8, 12, 19, and 22 after inoculation to track bacterial growth. In addition, to examine host response to either bacterial symbiont, triplicate controls of the axenic *C. sorokiniana* without initial DOM were grown in fresh COMBO medium. To track *C. sorokiniana* dynamics, 1 mL of each culture from Treatment F and the controls was collected and the relative fluorescence intensity unit (RFU) of Chlorophyll-*a* were measured as a proxy of microalgal density using a Synergy H1 microplate reader (Bio Tek, Winooski, VT, USA; excitation and emission wavelengths of 465 and 680 nm, respectively).

### Sample collection, DNA and RNA extraction, library preparation, and sequencing

The six tested bacterial isolates were identified using Sanger sequencing of the 16S rRNA gene. For DNA extraction, one colony of each isolate was resuspended in 90 µL 1 × PBS (0.2 µm filtered and autoclaved) and lysed by adding 5 µL lysozyme solution (50 mg/mL) and 100 µL Qiagen ATL buffer and incubated at 37°C for 30 min, followed by the protocol of the Qiagen DNeasy Blood & Tissue Kit (Qiagen, Hilden, Germany). The 16S rRNA gene was amplified using primers 27F (5′-AGAGTTTGATCMTGGCTCAG-3′) and 1492R (5′-TACGGYTACCTTGTTACGACTT-3′) (32). One microliters of template DNA was mixed with 10 µL of NEBNEXT 2× Master Mix (New England Biolabs, Ipswich, MA, USA) and 9 µL of Nuclease Free water. The PCR cycling conditions consisted of an initial denaturation at 95°C for 5 min, 25 cycles of denaturation at 95°C for 30 s, annealing at 55°C for 30 s, and extension at 72°C for 1 min, and a final extension step at 72°C for 5 min. The PCR products were stained with GelRed (Biotium, Fremont, CA, USA) and examined with gel electrophoresis using a 1% agarose gel. The qualified PCR products were sent out for PCR purification, quantification, and Sanger sequencing (Eurofins, Louisville, KY, USA). Low-quality bases were trimmed, and taxonomic classification was obtained using blastn (33) against the nucleotide collection (nr/nt) database available from National Center for Biotechnology Information (NCBI), with the best hit taxon used. Based on this analysis, the two representative symbionts used in this study were identified as *Curvibacter* sp. and *Roseomonas* sp. (but see below for reclassification based on genomic data).

After ensuring the two representative symbionts exhibited consistent growth patterns in large volume compared to those identified in the initial small volume experiments, the large volume experiment was repeated for collecting samples for transcriptomic analysis. This time, without subsampling at different times to track cell growth, each 100 mL culture was collected after 5 days of incubation in Treatment I and 12 days of incubation in Treatment F (Fig. 1). The incubation time was selected to capture optimal gene expression that can reflect the distinctive growth pattern of different interaction modes while also maintaining a sufficiently high cell density to yield enough RNA samples for sequencing. The collected cultures were filtered through 0.2 µm pore-size PES filters (Sigma Millipore, Burlington, MA, USA). For RNA extraction, cells were washed down from the filter and homogenized using QiaShredder Kits (Qiagen, Hilden, Germany). RNAs were extracted and purified using the RNeasy Micro Kit (Qiagen, Hilden, Germany) following the standard kit protocol. We quantified RNA using the Qubit RNA Broad Range Assay kit (Thermo Fisher Scientific, Waltham, MA, USA). The pure RNA samples were sent to the University of Michigan Center for Advanced Genomics to carry out library preparation, including rRNA depletion using QIAseq FastSelect 5S/16S/23S Kit and rRNA Plant Kits (Qiagen, Hilden, Germany) for samples containing bacteria and microalgae, respectively. Samples were sequenced using the NovaSeq S4 × 300 cycle platform (Illumina, San Diego, CA, USA) with 12.5% of the flow cell used, evenly distributed across all 15 samples.

To obtain a bacterial genomic reference for RNA mapping, whole-genome sequencing was conducted on the *Curvibacter* sp. and *Falsiroseomonas* sp. isolates. One colony of each isolate was resuspended in 90 µL 1 × PBS (0.2 µm filtered and autoclaved) and lysed by adding 5 µL lysozyme solution (50 mg/mL) and 100 µL Qiagen ATL buffer and incubated at 37°C for 30 min, followed by the protocol of the Qiagen DNeasy Blood & Tissue Kit Qiagen, Hilden, Germany. The extracted DNA was sent to the University of Michigan Center for Microbial Systems to carry out library preparation using plexWell Plus 24 Library Preparation Kit (seqWell, USA) and sequenced using an Illumina MiSeq 2 × 150 V2 flow cell with 30× coverage yield. Raw fastq files of RNA and whole genome sequencing were uploaded on NCBI sequence read archive with BioProject number PRJNA1266273.

### Bioinformatics

Raw bacterial whole-genome sequences and all RNA sequences were examined with fastqQC v0.12.1 (Andrews, 2010), and the adapters and low-quality base-pair (<20Q) were trimmed using Trimmomatic v0.39 (Bolger, Lohse, and Usadel 2014) with parameter “LEADING:3 TRAILING:3 SLIDINGWINDOW:4:20 MINLEN:36.” To prepare bacterial reference genomes for mapping, the trimmed whole-genome sequencing reads were *de novo* assembled using SPAdes v3.13.5 (34). Then, gene prediction was conducted using Prodigal (35). The completeness of bacterial reference genomes was examined using BUSCO v5.5.0 (36), indicating a completeness of 90.50% (database: *betaproteobacteria_odb10*) and 100% (database: *alphaproteobacteria_odb10*) for *Curvibacter* sp. and *Falsiroseomonas* sp., respectively. Bacterial taxonomy was identified with the Genome Taxonomy Database Toolkit (37) using the assembled contigs. At this time, bacterial taxonomy was assigned as *Curvibacter* sp. and *Falsiroseomonas* sp. Given whole genome sequences should provide a better resolution for classification than Sanger sequencing of 16S rRNA, we use the name *Falsiroseomonas* sp. instead of *Roseomonas* sp. in this study. This resulted in 6,985 and 6,780 identified genes from *Curvibacter* sp. and *Falsiroseomonas* sp., respectively (Table 1). For *C. sorokiniana*, we *de novo* assembled trimmed RNA-reads from across treatments (axenic, co-cultured with either bacteria) using Trinity (38). We removed rRNA reads using blastn (33) against the SILVA 138 database (39). Gene prediction was then conducted on the assembled contigs using TransDecoder (40). The resulting predicted coding sequences were compared to Chlorophyta protein sequences from NCBI (taxid: 3041) using blastp (33), and only those with matches were retained. The corresponding nucleotide sequences were then used as the reference transcriptome for mapping *C. sorokiniana* RNA-seq reads. Trimmed RNA reads were mapped to the reference genome/transcriptome using bowtie2 (41). The SAM output files were converted to BAM files, sorted, and indexed using samtools (42), for making transcript count tables using bedtools (43) that count only paired reads. The RNA read mapping rate for *C. sorokiniana* and both bacteria are shown in Table S1.

**TABLE 1:** Number of total genes and DE genes in bacteria and *C. sorokiniana* under different treatments^*a*^

	# of identified genes/transcripts	Total # of genes annotated as DOM transporter	Total # of genes annotated as part of a KEGG pathway	Experimental treatment comparison	# of DE genes/transcripts with higher relative abundance	# of DE genes/transcripts annotated to DOM transporter	# of DE genes/transcripts annotated to KEGG pathway
*Curvibacter* sp.	6,985	351	2,559	Treatment F	8	1	7
				Treatment I	295	10	138
*Falsiroseomonas* sp.	6,780	436	2,808	Treatment F	1,424	153	770
				Treatment I	1,571	166	925
*Chlorella sorokiniana*	76,416	N/A^*b*^	1,425	Add *Curvibacter* sp.	8,676	N/A	254
				Axenic	47	N/A	2
				Add *Falsiroseomonas* sp.	6,520	N/A	259
				Axenic	63	N/A	1
				Add *Curvibacter* sp.	8,592	N/A	250
				Add *Falsiroseomonas* sp.	6,546	N/A	260

^*a*^ DE genes were selected with adjusted *P*-value < 0.05 and abs(log2FoldChange) >=1.

^*b*^ N/A indicates analysis not applicable.

For functional annotation, predicted protein sequences were annotated using the KEGG database (44) using KAAS (KEGG Automatic Annotation Server) (45), where bacteria were annotated using the prokaryotes gene data set and *C. sorokiniana* using the “Green algae” gene data set (including *Chlamydomonas reinhardtii, Monoraphidium neglectum, Auxenochlorella protothecoides, Ostreococcus lucimarinus, Ostreococcus tauri, Micromonas commode, Micromonas pusilla*). In addition, we assigned functions to predicted protein sequences using the Clusters of Orthologous Groups (COG) (46) and Pfam databases (47) using the eggNOG-mapper (48). Specifically for *C. sorokiniana*, we limited annotations to the Chlorophyta taxonomic scope (taxid:3041), keeping annotations only from orthologs within this group.

### RNAseq data analysis

To examine the compositional distance of the gene expression profiles between treatments, a principal component analysis using variance stabilizing transformed transcript count tables was conducted separately for each species. The differentially expressed genes were also identified using DEseq2 (49) by comparing gene expression between Treatment F relative to Treatment I for bacteria. In this case, the log2 Fold Change (LFC) of each gene represents their relative expression level between treatments, where positive values indicate genes that were relatively more expressed under Treatment F, and negative value refers to genes that were relatively more expressed under Treatment I. The differentially expressed genes (DE genes) were genes with LFC ≥ 1 or ≤ −1 and adjusted *P*-value ≤ 0.05. For *C. sorokiniana*, the LFC values were calculated to compare the gene expression profiles of microalgae co-cultured with either bacterial symbiont relative to the axenic microalgal condition. Positive LFC values indicate that transcript expression was higher when co-cultured with the bacteria, and negative LFC values indicate that transcript expression was higher in the axenic state. The DE transcripts were transcripts with LFC ≥ 1 or ≤ −1 and adjusted *P*-value ≤ 0.05.

To examine which metabolic pathways were prioritized in the symbiont and host under different interaction modes, we used two approaches. First, we performed enrichment analysis, in the form of over-representation analysis with the “enrichKEGG” function from the R package “clusterProfiler” (50), to determine whether the observed over-representation of KEGG pathways (with DE genes) was significantly different from what would be expected by chance, using a hypergeometric test and all KEGG Orthology genes as the background. For this, adjusted *P*-values were calculated using the Benjamini-Hochberg method. In addition, the gene ratio was calculated as the number of DE genes in each KEGG pathway divided by the total number of DE genes assigned to any KEGG pathway. Second, we quantified the expression levels of DE genes related to key functional categories based on their relative abundance in rarefied count tables. The count tables were rarefied to the smallest sample size: 2,040,648 for bacteria and 16,076,584 for *C. sorokiniana*. For key functional categories, we explored bacterial responses to host-produced dissolved organic matter (DOM) under different interaction modes, where we examined DE genes annotated as “DOM-related transporters,” using a curated list of DOC (Dissolve Organic Carbon) transporters from Poretsky et al. (51) based on the COG database. Furthermore, to investigate potential metabolite exchange in phytoplankton-bacteria interactions, we also focused on DE genes in three metabolic pathways: biosynthesis of secondary metabolites, amino acid metabolism, and metabolism of cofactors and vitamins. For this, KO numbers for each DE genes assigned from KAAS were assigned to metabolic pathways using the R package KEGGREST (52).

To compare gene abundance between treatments, we used the Wilcoxon rank-sum test. Raw *P*-values were adjusted for multiple testing using the Benjamini-Hochberg method, and resulting adjusted *P*-value ≤ 0.1 was used to determine significance. Given the limited number of replicates (*n* = 3), we used a relaxed threshold of *P* ≤ 0.1 to reduce the risk of false negatives, while noting this increases the risk of false positives. Results should, therefore, be interpreted with caution. All analyses were performed in R (version 4.3.2, R Core Team, 2022). The R scripts, original data, and annotation results are provided on Github: https://github.com/jinnyyang/Chlorella_Symbionts_InteractionModes.

## RESULTS

### Bacterial and host growth dynamics in batch culture

We selected six bacteria isolated from *C. sorokiniana* based on their distinctive colony morphology to enable easy tracking of growth of each culture when in co-culture and tested their growth response when constraining conditions to only allow for a unidirectional innate host selection (with host innate DOM but no host cells; Treatment I) and to promote a bidirectional host-microbe feedback (with host cells after removal of previously produced host innate DOM; Treatment F). Among the six isolates, we found that a *Curvibacter* sp. and *Falsiroseomonas* sp. showed the most distinct growth patterns under these two treatments in a 50 mL volume (Fig. S1). *Curvibacter* sp. exhibited a higher density in Treatment I than Treatment F by day 9. On the contrary, *Falsiroseomonas* sp. was the only isolate that maintained a lower density under Treatment I than Treatment F throughout the experiment (12 days). We, therefore, took *Curvibacter* sp. to represent a symbiont favored by innate host selection and *Falsiroseomonas* sp. to represent a symbiont favored by host-microbe feedback.

These two symbionts showed similar distinct growth patterns between the two treatments when grown in a 100 mL volume (Fig. 1). *Falsiroseomonas* sp. preferred the Treatment F and leveled off at Day 19, and *Curvibacter* sp. preferred Treatment I, regardless of the mono- or mixed-bacterial culture conditions (Fig. 1A through D). Yields remained consistent at the time of sample collection when we repeated the same experiment for collecting biomass for transcriptomics (Fig. S2). For *C. sorokiniana*, there was no difference in population dynamics between axenic status and when co-cultured with *Curvibacter* sp. On the other hand, when co-cultured with *Falsiroseomonas* sp. or the mix of both bacterial species, *C. sorokiniana* growth leveled off at lower density at Day 15 compared to the axenic status (Fig. 1E).

### Overall gene expression profiles of symbionts and host among treatments

**Fig 2:**
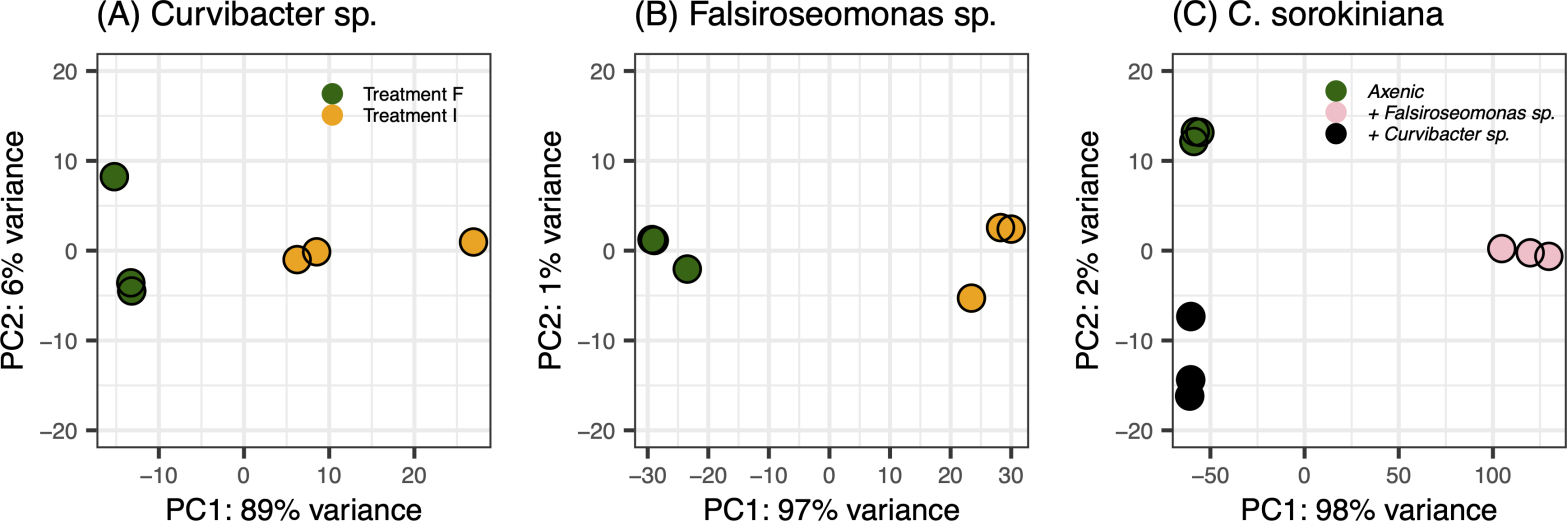
Principal component analysis revealing the gene expression profile differences between treatments for (**A**) *Curvibacter* sp., (**B**) *Falsiroseomonas* sp., and (**C**) *C. sorokiniana*. For both bacteria, green and yellow points indicate gene expression profiles under Treatment F and Treatment I, respectively. For *C. sorokiniana*, the green, pink, and black points represent the axenic state when co-cultured with *Falsiroseomonas* sp. and when co-cultured with *Curvibacter* sp*.* The percentages in the *X* and *Y* axis labels show the proportion of variation explained by the first and second PC.

We explored the variability in overall gene expression profiles of both bacterial symbionts and hosts across treatments by conducting a Principal Component Analysis on the variance-stabilized transformed transcript count table. We found a clear clustering based on *Treatment I* and *F* for both bacteria, with 89% and 97% variance explained by the first PC axis for *Curvibacter* sp. and *Falsiroseomonas* sp., respectively (Fig. 2A and Fig. 2B). By pairwise comparing the gene expression profile differences (Euclidean distance, including both number of DE genes and effect size of change), we found that *Falsiroseomonas* sp. showed a significantly larger difference between Treatment I and F than *Curvibacter* sp. (Fig. S3A). For *C. sorokiniana*, the pair-wise comparison among treatments showed that the difference between co-cultured with *Falsiroseomonas* sp. vs axenic status was larger than the other two comparisons: between co-cultured with *Curvibacter* sp. vs axenic and between co-cultured with either symbiont (Fig. S3B). This is despite a slightly smaller number of DE transcripts, the lower number being compensated by the larger effect size of the changes.

**Fig 3:**
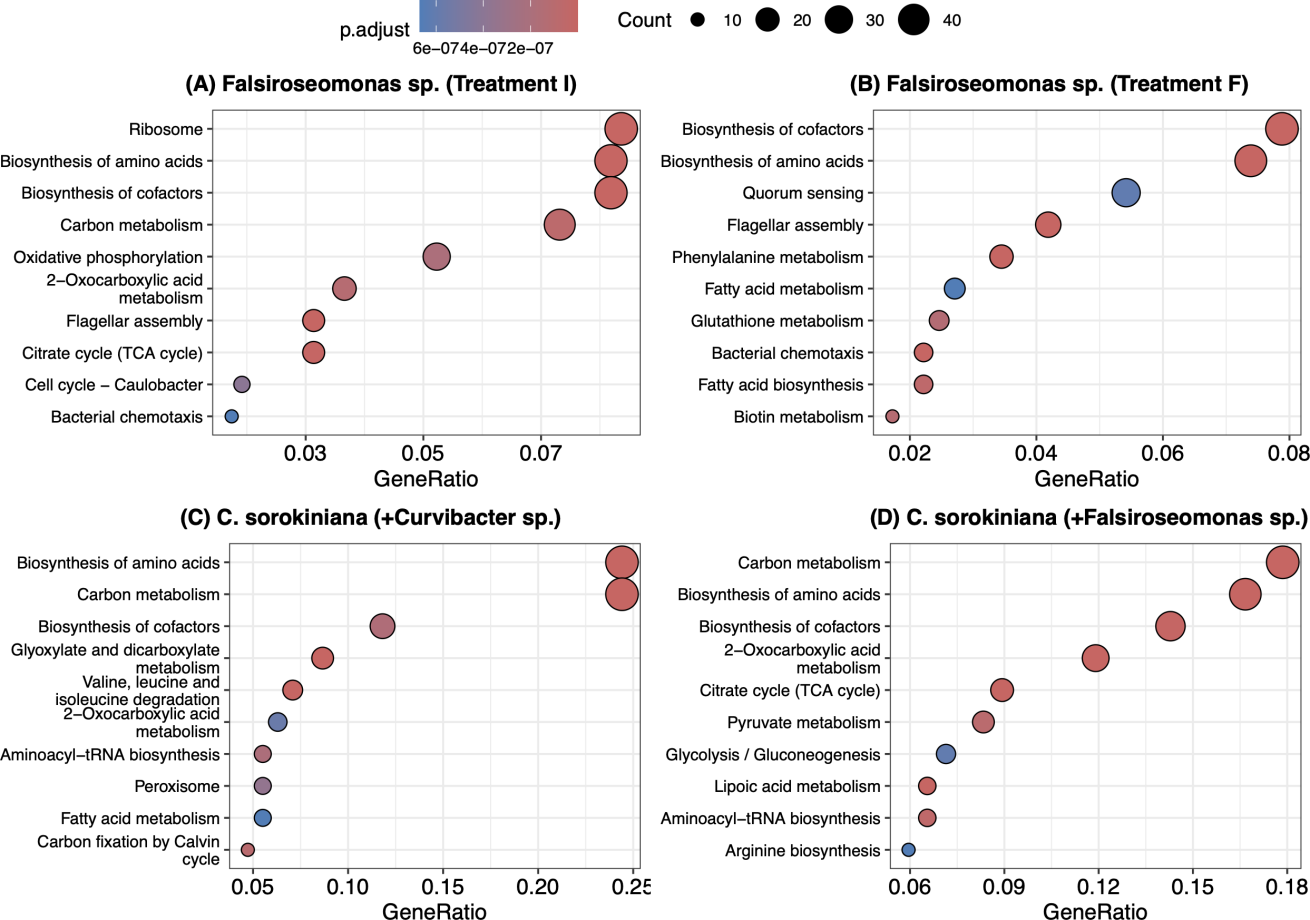
Enrichment analysis on KEGG pathway-annotated DE genes. (**A**) The top 10 enriched pathways in *Falsiroseomonas* sp*.* based on DE genes relatively more expressed under Treatment I and (**B**) Treatment F, as well as in (**C**) *C. sorokiniana* based on DE genes relatively more expressed when co-cultured with *Curvibacter* sp. and (**D**) co-cultured with *Falsiroseomonas* sp., compared to their corresponding axenic states, respectively. Gene ratios were calculated by dividing the number of DE genes assigned to each pathway by the total number of KEGG annotated DE genes in that treatment. The point size indicates the number of DE genes assigned to each pathway. The color of points indicates the adjusted *P*-value to determine the observed number of DE genes in a given pathway significantly higher than expected by chance (adjusted-*p* ⩽ 0.05).

### Number of differentially expressed genes between treatments of symbionts and host

For bacteria, we compared the number of differentially expressed (DE) genes between Treatment F and Treatment I (Table 1). For *Curvibacter* sp.*,* 6,985 genes were identified in the genome and 8 and 295 DE genes were more highly expressed under Treatment F and Treatment I, respectively. For *Falsiroseomonas* sp*.*, among a total of 6,780 genes identified in the genome, 1,424 and 1,571 of DE genes were more highly expressed under Treatment F and Treatment I, respectively. For *C. sorokiniana*, a total of 76,416 transcripts remained after filtering the *de novo* assembly. When comparing its relative transcript levels when co-cultured with either bacteria to its axenic state, we found 8,676 and 6,520 DE transcripts more highly expressed when co-cultured with *Curvibacter* sp. and *Falsiroseomonas* sp.*,* respectively. When comparing the relative expression levels of *C. sorokiniana* between co-cultured with *Curvibacter* sp. and with *Falsiroseomonas* sp., we observed 8,592 and 6,546 DE transcripts were more highly expressed DE transcripts, respectively, again including uniquely detected transcripts in each condition.

### Enrichment analysis on symbionts between treatment I and F

Among the KEGG-annotated DE genes in *Curvibacter* sp., we identified 1 enriched pathway, Oxidative phosphorylation, based on 7 DE genes under Treatment F, and 13 enriched pathways based on 138 DE genes under Treatment I. Among these enriched pathways, the top 10 were ribosome, carbon metabolism, biosynthesis of amino acids, oxidative phosphorylation, glyoxylate and dicarboxylate metabolism, 2-oxocarboxylic acid metabolism, butanoate metabolism, pyruvate metabolism, valine, leucine and isoleucine biosythesis, and citrate cycle (Table 1; Fig. S4A). For *Falsiroseomonas* sp., 45 enriched pathways were found among 770 KEGG-annotated DE genes under Treatment F, and 47 enriched pathways among 925 DE genes under Treatment I (Fig. S4B and S4C). Among the top 10 enriched pathways in *Falsiroseomonas* sp., biosynthesis of cofactors, biosynthesis of amino acids, flagellar assembly, and bacterial chemotaxis were consistently enriched in both treatments (Fig. 3A and B). By contrast, ribosome function, carbon metabolism, oxidative phosphorylation, 2-oxocarboxylic acid metabolism, and the citrate cycle were only enriched under Treatment I (Fig. 3A), while quorum sensing, phenylalanine metabolism, fatty acid metabolism, glutathione metabolism, and biotin metabolism were only observed as an enriched function under Treatment F (Fig. 3B).

### Expression level of DOM-related transporters annotated DE genes in symbionts

**Fig 4:**
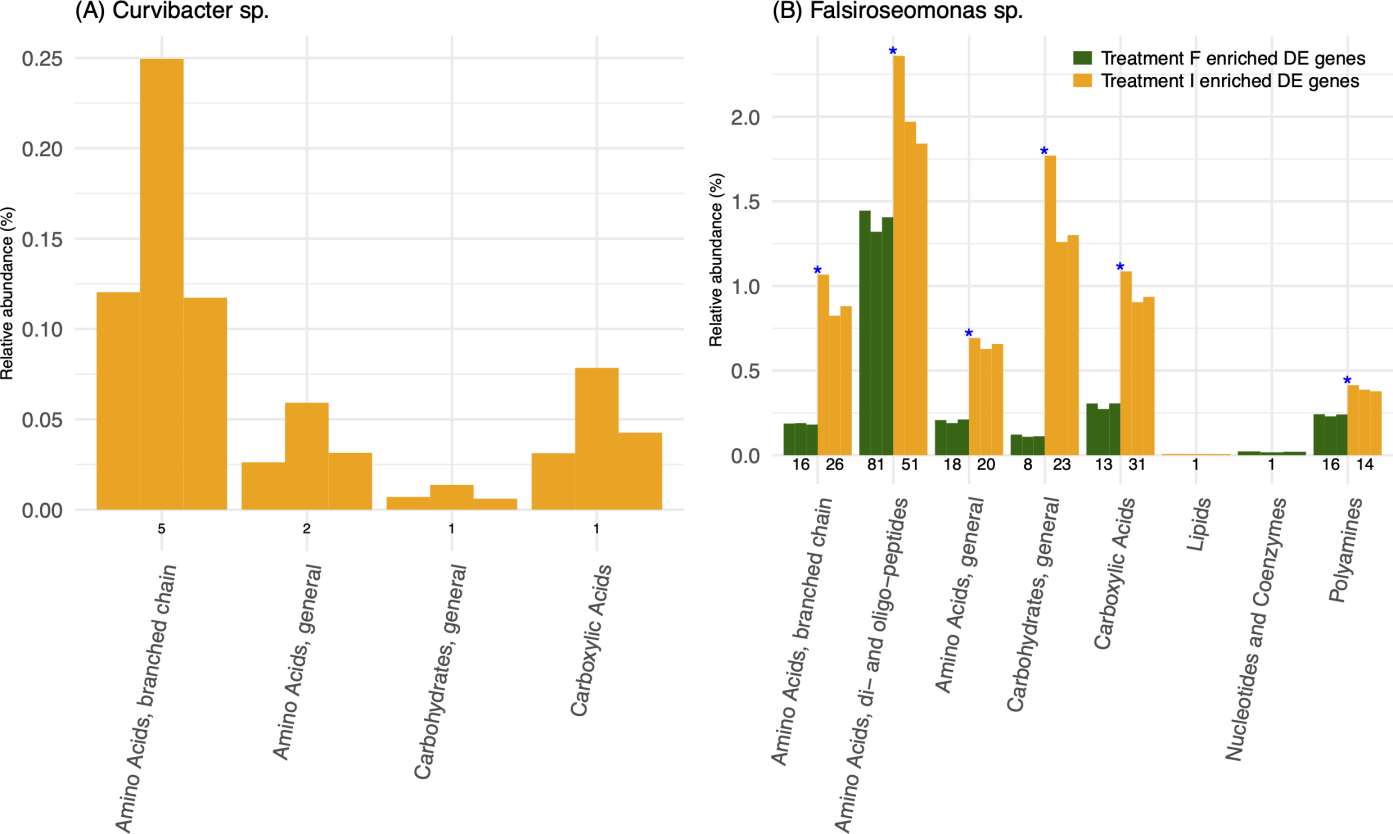
The relative abundance of DOM-transporter annotated DE genes of (**A**) *Curvibacter* sp. and (**B**) *Falsiroseomonas* sp. under Treatment I and F. Each colored bar represents a replicate for the indicated treatment (three replicates per treatment). Green bars show DOM-transporter annotated DE genes with higher relative abundance under Treatment F, and yellow bars indicate DOM-transporter annotated DE genes with higher relative abundance under Treatment I. Numbers below bars are the number of annotated DE genes in each transporter functional category. Both treatments were performed in triplicate. Blue asterisks denote significant differences in relative abundance between treatments based on a Wilcoxon test with adjusted *P*-values ≤ 0.1.

Considering the important role of algal-produced DOM and bacterial reliance on it for growth, we focused on DE genes that were annotated as DOM-related transporters (based on COG database) under two treatments of either bacteria. The main finding was a much higher number of DE genes in this category for *Falsiroseomonas* sp. compared to *Curvibacter* sp. (Table 1). For both symbionts, the largest number of DE transporters was involved in amino acid of peptide transport, and both the number and effect size of the change was higher under Treatment I than F (Fig. 4; Table 1). Furthermore, when considering the relative expression ratio, based on the proportion of the relative abundance, we found a higher proportion on “Carbohydrates, general” in Treatment I than F for both symbionts (Fig. S5). For *Falsiroseomonas* sp., categories “Amino Acids, di- and oligo-peptides,” “Polyamines,” “Nucleotides and Coenzymes” were higher in Treatment F than I.

### Expression level of KEGG annotated DE genes in symbionts

When focusing on the relative abundance for KEGG annotated DE genes in amino acid metabolism, and metabolism of cofactors and vitamins, we typically observed a higher relative abundance in Treatment I than F for both symbionts (Fig. S6 and S7). Notable exception in *Falsiroseomonas* sp. where we observed pathways related to defense that had a higher relative transcript abundance under Treatment F than under Treatment I. These included the biosynthesis of secondary metabolites such as novobiocin, phenazine, prodigiosin, and tropane, piperidine, and pyridine alkaloids (Fig. S7A); amino acid metabolism pathways including histidine metabolism and valine, leucine, and isoleucine biosynthesis (Fig. S7B); and vitamin and cofactor metabolism pathways such as biotin metabolism and ubiquinone and other terpenoid-quinone biosynthesis (Fig. S7C).

### Enrichment analysis for *C. sorokiniana* when co-cultured with either symbiont

Among the KEGG-annotated DE genes in *C. sorokiniana*, co-culture with *Curvibacter* sp. resulted in 254 DE genes relative to axenic conditions corresponding to 37 enriched pathways, while 259 genes corresponding to 47 enriched pathways were more expressed in co-cultured with *Falsiroseomonas* sp. compared to axenic conditions (Fig. S8). Among the top 10 enriched pathways when *C. sorokiniana* was co-cultured with either symbiont were the biosynthesis of amino acids, carbon metabolism, biosynthesis of cofactors, aminoacyl-tRNA biosynthesis, and 2-oxocarboxylic acid metabolism. However, glyoxylate and dicarboxylate metabolism, valine, leucine, and isoleucine degradation, peroxisome function, fatty acid metabolism, and carbon fixation by the Calvin cycle were exclusively observed when co-cultured with *Curvibacter* sp. (Fig. 3C), whereas the citrate cycle (TCA cycle), pyruvate metabolism, glycolysis/gluconeogenesis, lipoic acid metabolism, and arginine biosynthesis were only detected when co-cultured with *Falsiroseomonas* sp. (Fig. 3D)

### Expression level of KEGG annotated DE genes in *C. sorokiniana* when co-cultured with either symbiont

**Fig 5:**
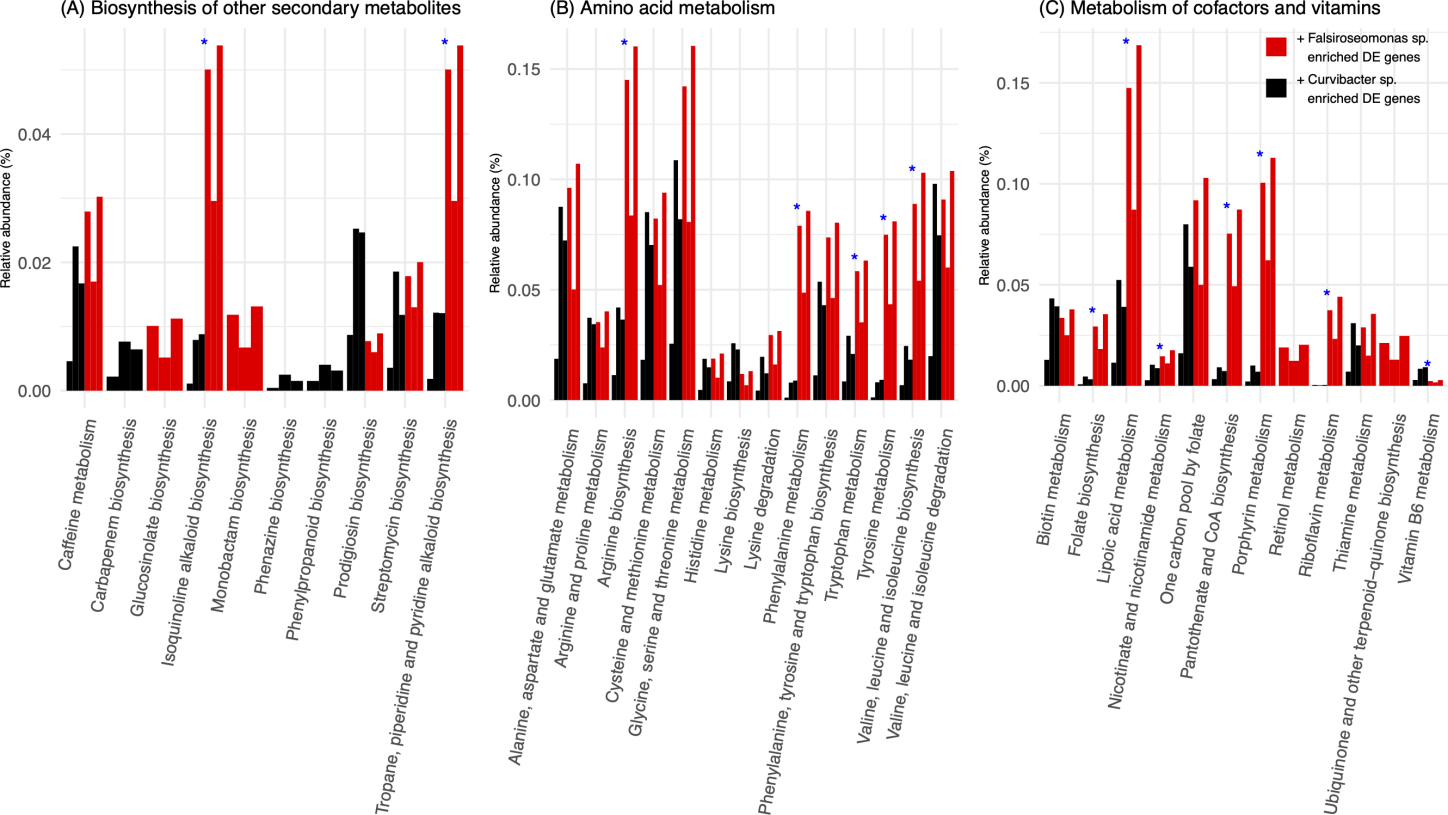
The relative abundance of KEGG pathway-annotated differentially expressed (DE) genes in *C. sorokiniana* across three KEGG categories: (**A**) biosynthesis of secondary metabolites, (**B**) amino acid metabolism, and (**C**) metabolism of cofactors and vitamins. Each colored bar represents a replicate for the indicated treatment (three replicates per treatment). Black and red bars representing the relative abundance of DE genes were more expressed when co-cultured with *Curvibacter* sp. (black) and *Falsiroseomonas* sp. (red) compared to their axenic states. Blue asterisks denote significant differences in relative abundance between treatments based on a Wilcoxon test with adjusted *P*-values ≤ 0.1.

The biosynthesis of secondary metabolites, isoquinoline alkaloid biosynthesis, as well as tropane, piperidine, and pyridine alkaloid biosynthesis, were significantly more expressed when *C. sorokiniana* was co-cultured with *Falsiroseomonas* sp., whereas phenylpropanoid biosynthesis was exclusively differentially expressed during co-culture with *Curvibacter* sp. (Fig. 5A). For amino acid metabolism, the metabolism of arginine, phenylalanine, tryptophan, tyrosine, and valine, leucine, and isoleucine was more expressed in the presence of *Falsiroseomonas* sp. (Fig. 5B). Finally, for the metabolism of cofactors and vitamins, folate biosynthesis, lipoic acid metabolism, nicotinate and nicotinamide metabolism, pantothenate and CoA biosynthesis, porphyrin metabolism, and riboflavin metabolism were more expressed when co-cultured with *Falsiroseomonas* sp., while vitamin B6 metabolism was more expressed during co-culture with *Curvibacter* sp. (Fig. 5C). We notice that some pathways shown in Fig. 5 are not known to occur in *C. sorokiniana* which may instead suggest the presence pathway of related compounds, but we cannot determine this based on these data alone. Nevertheless, these results indicate that different metabolic pathways are prioritized in *C. sorokiniana* depending on the symbiotic partner, highlighting changes not only in overall general metabolic pathways but also in the three sets of notably differentially expressed pathways involved in host-microbe interactions.

## DISCUSSION

Host-microbiome interactions play a critical role in microbiome community assembly, determining which functional groups of microbiota can establish within the microbiome as well as the functions that the microbiome provides to the host organism (9, 53, 54). We previously showed how innate host control and host-microbiome feedback led to different microbiome compositions at the community level and acted simultaneously in sustaining microbiome diversity (17). This finding inspired our hypothesis that the different microbiomes emerging in function of the presence of host-produced DOM vs live host cells were driven by symbionts with contrasting abilities in adjusting their growth and metabolic status in response to unidirectional innate host selection vs bidirectional host-microbe feedback. Our new results provide insights into what underpins these observations from single microbial symbiont-host interactions using two contrasting symbionts that we isolated from the microbiome of *C. sorokiniana*, *Curvibacter* sp., and *Falsiroseomonas* sp., both of which are reported in phytoplankton microbiomes (55–58).

### Contrasting symbiont responses to different host-microbe interaction modes

By examining both physiological responses and gene expression profiles, we observed contrasting patterns in growth, competition outcomes, and transcriptional shifts between *Curvibacter* sp. and *Falsiroseomonas* sp. under uni- and bidirectional host-microbe interactions. *Curvibacter* sp. showed higher fitness when provided only with host-derived DOM and exhibited a smaller shift in gene expression in the presence of host cells. In contrast, *Falsiroseomonas* sp. displayed larger shifts in gene expression and higher fitness when co-cultured with host cells. These findings support our hypothesis that *Curvibacter* sp. can utilize host-derived dissolved organic matter (DOM) without needing direct interactions with host cells. In contrast, *Falsiroseomonas* sp. dominated under bidirectional interactions, showing significant gene expression shifts from unidirectional conditions, supporting its potential to directly interact with the host to its benefit. This was further supported by our observation of how general metabolic pathways were prioritized under different host-microbe interaction modes in *Falsiroseomonas* sp., where prioritized pathways related to host-microbe interaction in the presence of the host, but focused more on carbon metabolism when only host DOM was available (Fig. 3A and B).

### Shift in DOM-related transporters annotated DE genes in symbionts between different host-microbe interaction modes

**Fig 6:**
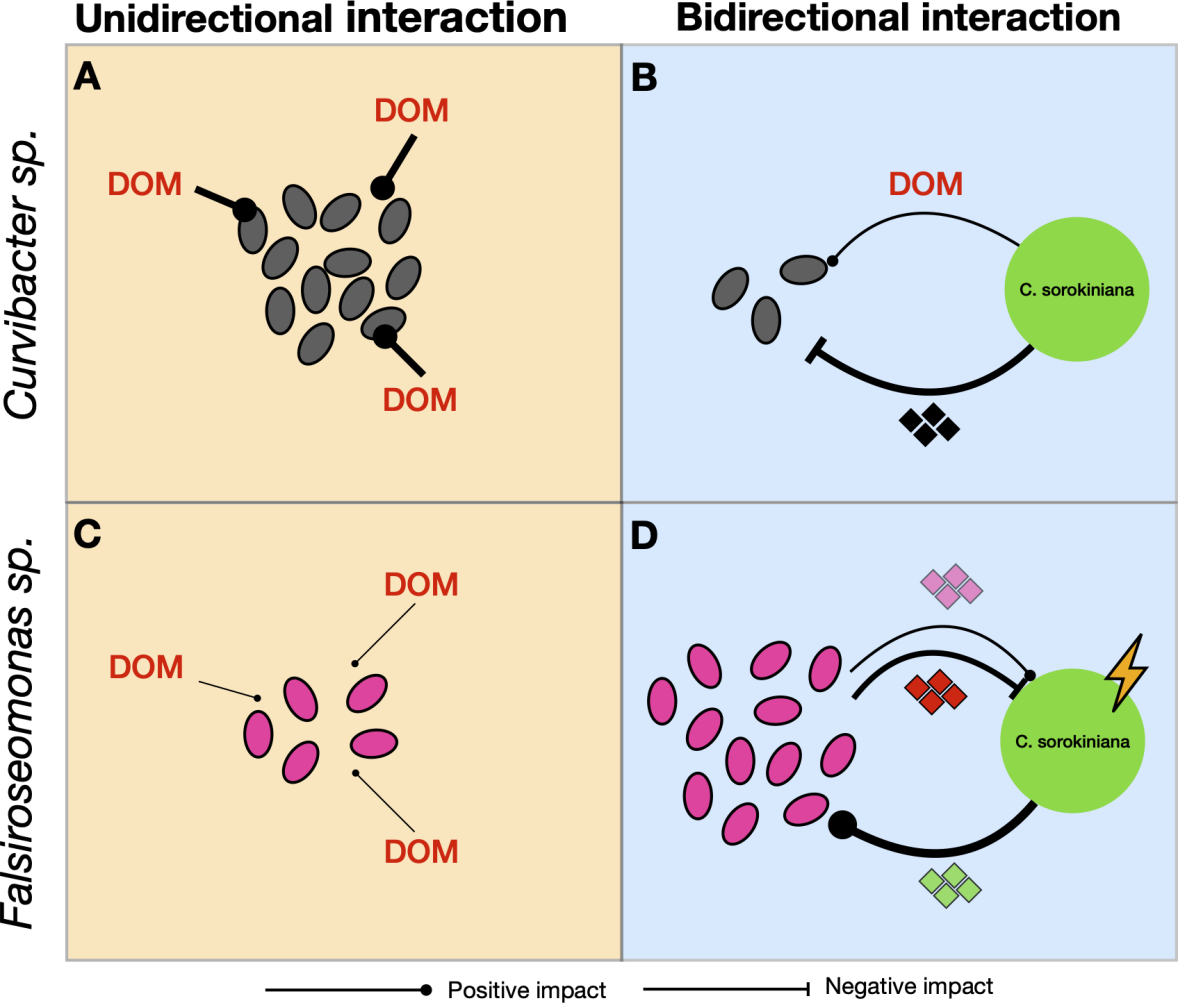
Conceptual figure illustrating the hypotheses stemming from growth responses and transcriptomics data. The thickness of arrows represents the strength of impact on recipient growth. (**A**) *Curvibacter* sp. thrives in the unidirectional interaction mode, benefiting from host-produced dissolved organic matter (DOM), (**B**) but lacks the ability to engage in feedback interactions with the host. In addition, we found that a decrease of *Curvibacter* sp. may be caused by antibacterial metabolites produced by the host (black rhombus), resulting in lower abundance under bidirectional conditions even though the host continued to produce DOM. (**C**) *Falsiroseomonas* sp. exhibits relatively limited growth under unidirectional interactions due to its lower ability to use axenically produced host DOM (**D**) but can drive direct interactions with the host under a bidirectional interaction mode. Such bidirectional interactions likely involve symbiont-produced metabolites that can be beneficial to the host (pink rhombus, e.g., vitamins) or antagonistic (red rhombus, e.g., algicidal secondary metabolites). These interactions trigger shifts in host metabolism and the subsequent release of specific host metabolites (green rhombus, e.g., nitrogen-rich amino acids, arginine) that benefit the symbiont. In our case, host growth was reduced during bidirectional interaction with *Falsiroseomonas* sp., likely because its antagonistic impact outweighed the beneficial effects on the host.

The exchange of metabolites plays an important role in phytoplankton-bacteria interactions (14, 59), with DOM transporter gene expression in symbionts reflecting their response to changes in phytoplankton-derived DOM composition or difference in their capacity to use them. In this study, we found that both symbionts showed an overall higher number and higher relative expression of DE DOM transporter genes when only host DOM was available compared to when host cells were present (Fig. 4; Fig. 6A and Fig. 6C). Increased expression could be a response to increased presence or the opposite, a response to DOM limitation, which may be expected to arise as DOM cannot be replenished in the axenic DOM-only treatment. The proportional shift in transporter categories (Fig. S5) suggests that symbionts prioritized different transporters in response to host DOM composition changes between the two host-microbe interaction modes. The unidirectional host-microbe interaction corresponded with transporters for carbon-rich host DOM (e.g., Carbohydrates), whereas the increased presence of nitrogen-rich transporter expression of *Falsiroseomonas* sp. in the presence of host cells suggests that this symbiont induces the release of nitrogen-rich host DOM (e.g., Amino acids) through bi-directional interactions with the host in Treatment F. Again, these patterns could alternatively be interpreted as more carbon limitation in the Treatment I and more nitrogen limitation in the Treatment F. However, since we did not measure carbon and nitrogen content in the media, we cannot determine which interpretation is more likely. Nonetheless, these findings are consistent with our hypothesis that *Falsiroseomonas* sp. is able to direct changes to its host’s DOM composition; yet, direct metabolomic profiling is needed to validate shifts in DOM composition.

### Contrasting responses in *C. sorokiniana* co-cultured between different symbionts

Contrasting responses of symbionts when co-cultured with *C. sorokiniana* host cells were accompanied by distinct host reactions when co-cultured with either symbiont. Compared to the axenic condition, *C. sorokiniana* growth remained unchanged and with a smaller shift in gene expression profile when co-cultured with *Curvibacter* sp. but decreased in growth and a large shift in expression profile when co-cultured with *Falsiroseomonas* sp. (Fig. 2E and 3; Fig. S2). Although the number of DE transcripts was higher with *Curvibacter* sp., the effect size of expression changes (Fig. 2; Fig. S3) and number of uniquely expressed transcripts (Fig. S8) were larger in presence of *Falsiroseomonas* sp. These results suggested that the *Falsiroseomonas* sp. triggered a high number of uniquely expressed transcripts, whereas co-culturing with *Curvibacter* sp. enhanced the expression of many transcripts that were already present in the axenic state. While the host clearly responds to the presence of *Curvibacter* sp. as well, the larger overall shifts in gene expression, and the results from the bacterial response between treatments, are still partially supportive of our hypothesis that only *Falsiroseomonas* sp. was able to bring a strong impact on the host gene expression through bidirectional host-microbe interaction.

The responsive shifts in growth and gene expression between *C. sorokiniana* and *Falsiroseomonas* sp. indicate a direct phytoplankton-bacteria feedback interaction. When focusing on potential pathways that were prioritized during bidirectional host-microbe feedback modes, we found many pathways previously involved in phytoplankton or plant-microbe interaction that were among the top 10 enriched in *Falsiroseomonas* sp. only when co-cultured with the host (Fig. 3B). These included quorum sensing (60), phenylalanine metabolism (15, 61), fatty acid metabolism (62), and biotin metabolism (63) (Fig. 3B). This suggests that *Falsiroseomonas* sp. may initiate communication with the host by producing signaling molecules and metabolites. In addition, we observed increased metabolism of biotin and ubiquinone and other terpenoid-quinone biosynthesis (which may involve in biosynthesis of Vitamin K2) in *Falsiroseomonas* sp. when co-cultured with the host (Fig. S7; Fig. 6D), suggesting a potential beneficial impact on the host. However, co-cultured with *C. sorokiniana* appears to trigger potential defense-related metabolic pathways against algae in *Falsiroseomonas* sp. as well, including phenazine and prodigiosin biosynthesis (64, 65) (Fig. S7).

On the side of *C. sorokiniana*, we observed high expression of nitrogen-rich amino acid (arginine biosynthesis and phenylalanine/tryptophan metabolism) and vitamin-related pathways (e.g., folate, porphyrin, pantothenate, and riboflavin) when co-cultured with *Falsiroseomonas* sp. (Fig. 5). This suggests that the host may respond to *Falsirosemonas* sp. by activating nitrogen metabolism, potentially benefiting the bacterium by enhancing nitrogen recycling and producing amino acids or nitrogenous metabolites that it can utilize (Fig. 6D). The underlying mechanisms require further investigation, particularly to determine whether this interaction involves nitrogen-rich metabolites exchange. Nevertheless, these findings support that the bi-directional interaction between *C. sorokiniana* and *Falsiroseomonas* sp. remodels the metabolic status in both symbiotic partners.

Although *C. sorokiniana* growth was reduced when co-cultured with *Falsiroseomonas* sp., the density of *Falsiroseomonas* sp. stabilized by Day 19 after the leveling off of *C. sorokiniana* on Day 15 (Fig. 3). This suggests that *Falsiroseomonas* sp. is dependent on and responsive to *C. sorokiniana* for its growth. This interpretation is consistent with transcriptomic analysis, which revealed that *Falsiroseomonas* sp. exhibits both potentially beneficial and defensive mechanisms toward *C. sorokiniana*, adopting a parasitic symbiotic role by relying on the host while also contributing to its reduced growth.

Furthermore, although *Curvibacter* sp. exerted a relatively smaller influence on the metabolic state shifts of *C. sorokiniana*, we observed differential expression of many amino acids, vitamins, and secondary metabolites in *C. sorokiniana* co-cultured with *Curvibacter* sp. compared to the axenic status. In addition, pathways of phenylpropanoid biosynthesis that are known as producing antimicrobial metabolites (e.g, coumarins) by plants (66) were differentially expressed only in co-cultures with *Curvibacter* sp. (Fig. 5A). This selective expression could explain the reduced growth of *Curvibacter* sp. when co-cultured with *C. sorokiniana* cells (Fig. 2) and suggests that the host differentially modulates bacterial populations in response to distinct symbionts. These findings further demonstrated that, although *Curvibacter* sp. has no substantial impact on *C. sorokiniana* growth, it triggers the production of potential antibacterial metabolites, resulting in a measurable shift in the host’s gene expression profile (Fig. 6B). As such, these data are opposed to our hypothesis, in that both symbionts have a bidirectional interaction with the host. However, for *Falsiroseomonas* sp., these are feedback interactions that improve conditions for the symbiont. In contrast, with *Curvibacter* sp., the host response is less likely to be driven by the symbiont in its own interest. Although it remains unclear whether this is a specific response to *Curvibacter* sp. or a generic response to any bacterium that does not have the ability to modulate the physiology of its host to its own benefit.

### Caveats

We collected Treatment I at day 5 and Treatment F, along with the axenic control, at day 12. This approach aimed to capture the gene expression profiles during the most active phase of host-microbe interactions while also providing enough bacterial biomass for RNA sequencing. However, we acknowledge that this can be a concern especially for *Curvibacter* sp. which was collected during the exponential growth phase in Treatment I, while it was in the beginning of the death phase in Treatment F (Fig. 1). Therefore, our observations for *Curvibacter* sp. gene expression shifts between treatments could be due to shifts in growth phase in addition to the host-microbe interactions we aimed to study. Nevertheless, the magnitude of the Curvibacter sp. gene expression shifts remained smaller compared to those observed for *Falsiroseomonas* sp., which were collected at exponential growth phase in Treatment I and F (Fig. 2). Hence, we argue that our comparison of the different interaction modes remains valid. Yet, further analysis of gene expression across different growth phases and time points, for example, collected at Day 5 and 12 for both treatments, would provide a more comprehensive examination.

Treatment I contained axenic host spent medium without live host cells, while Treatment F included live host cells with their initially produced DOM removed. This design aimed to capture direct host-microbe interactions without the influence of initial host DOM in a bidirectional host-microbe interaction setting. However, we did not obtain DOM quantification data and acknowledge that the observed shifts in growth and gene expression may be affected by the amount of DOM in addition to changes in DOM composition resulting from different host-microbe interactions. For example, higher growth of *Falsiroseomonas* sp. in Treatment F than I can be because of the continuous DOM production with presence of host cells in Treatment F while its growth was lower by the limited DOM in Treatment I. Despite this, we observed substantial differences in gene expression and metabolic pathway activation between treatments, along with distinct impacts on the host. We argue that these shifts in growth and gene expression were primarily driven by changes in host-microbe interaction modes. Additionally, the absence of DOM composition data limited our ability to identify specific compounds responsible for the different interaction modes. Nevertheless, our transcriptomic analysis provides important insights into potential mechanisms for future metabolomics studies.

### Conclusions

Our study demonstrated how host and symbionts shift in their growth and metabolic status between two host-microbe interaction modes. These dynamics lead to distinct outcomes in the competition outcome between symbionts and influence the process of microbiome assembly. Consistent with previous studies, host-microbe interactions remodel growth responses and gene expression in both host and symbiont. Previously, this has mostly been studied by investigating the bacterial response to added phytoplankton metabolites or comparisons between axenic and co-cultured treatments of phytoplankton, both only focusing on one side of the interaction (15, 16, 27). Our work advanced the field by simultaneously investigating impacts of host-microbe interaction modes by contrasting unidirectional interaction driven solely by host innate DOM with bidirectional host-microbe feedback, and the impacts with different symbionts involved, on both recipients.

## SUPPLEMENTAL MATERIAL

Supplemental material msystems.00803-25-s0001.docx Supplemental figures and table.

ASM does not own the copyrights to Supplemental Material that may be linked to, or accessed through, an article. The authors have granted ASM a non-exclusive, world-wide license to publish the Supplemental Material files. Please contact the corresponding author directly for reuse.
