# Supplementary material for: Dissecting two contrasting phytoplankton-symbiont interaction modes based on population dynamics and gene expression patterns
Source: mSystems. 2025 Nov 4;10(12):e00803-25. doi: 10.1128/msystems.00803-25 (PMC12710304; doi:10.1128/msystems.00803-25)
Supplement: Supplemental material — Supplemental figures and table. [file msystems.00803-25-s0001.docx]

**Dissecting two contrasting phytoplankton-symbiont interaction modes based on population dynamics and gene expression patterns**

Jinny Wu Yang^1,2^ and Vincent J Denef^1^

^1^Ecology and Evolutionary Biology, University of Michigan, Ann Arbor, MI, USA

^2^Plant Resilience Institute, Michigan State University, East Lansing, MI, USA

Corresponding authors:

Jinny Wu Yang

Plant Resilience Institute

Michigan State University, East Lansing, MI, USA

Email: yangjinn@msu.edu

Vincent Denef

Department of Ecology and Evolutionary Biology

University of Michigan, Ann Arbor, MI, USA

Email: vdenef@umich.edu

**Supplementary information**

**Table S1.** Number of RNA reads during quality filtration and mapped to genome/transcriptome references.

| Sample | Treatment | Organisms in treatment | Replicates | # of reads (paired) | | # of reads (paired) mapped to | | |
| --- | --- | --- | --- | --- | --- | --- | --- | --- |
|  |  |  |  | Raw | After Trimmomatic | *Curvibacter* sp. reference genome | *Falsiroseomonas* sp. reference genome | *C. sorokiniana* reference transcripts |
| FW1 | Treatment F | *Curvibacter sp. + C. sorokiniana* | 1 | 84,361,333 | 77,324,259 | 2,040,648 |  | 20,287,198 |
| FW2 |  |  | 2 | 94,596,164 | 84,976,931 | 1,0769224 |  | 25,005,664 |
| FW3 |  |  | 3 | 93,2796,34 | 81,488,456 | 9,303,726 |  | 26,967,212 |
| IW1 | Treatment I | *Curvibacter sp.* | 1 | 74,400,918 | 64,386,707 | 40,638,620 |  |  |
| IW2 |  |  | 2 | 78,506,118 | 67,514,569 | 31,854,556 |  |  |
| IW3 |  |  | 3 | 80,464,003 | 69,378,257 | 36,742,556 |  |  |
| FP1 | Treatment F | *Falsiroseomonas sp. + C. sorokiniana* | 1 | 91,403,132 | 84,277,295 |  | 21,072,188 | 33,522,874 |
| FP2 |  |  | 2 | 77,531,188 | 70,930,012 |  | 7,731,894 | 19,206,502 |
| FP3 |  |  | 3 | 86,959,170 | 78,742,058 |  | 24,444,320 | 34,056,764 |
| IP1 | Treatment I | *Falsiroseomonas sp.* | 1 | 46,961,595 | 40,022,387 |  | 9,903,548 |  |
| IP2 |  |  | 2 | 74,520,808 | 65,921,965 |  | 21,803,706 |  |
| IP3 |  |  | 3 | 81,784,277 | 71,331,418 |  | 26,602,038 |  |
| F1 | Axenic host | *C. sorokiniana* | 1 | 89,067,002 | 81,370,241 |  |  | 20,402,884 |
| F2 |  |  | 2 | 93,316,408 | 84,288,970 |  |  | 17,599,514 |
| F3 |  |  | 3 | 93,100,371 | 85,251,645 |  |  | 16,076,584 |

**
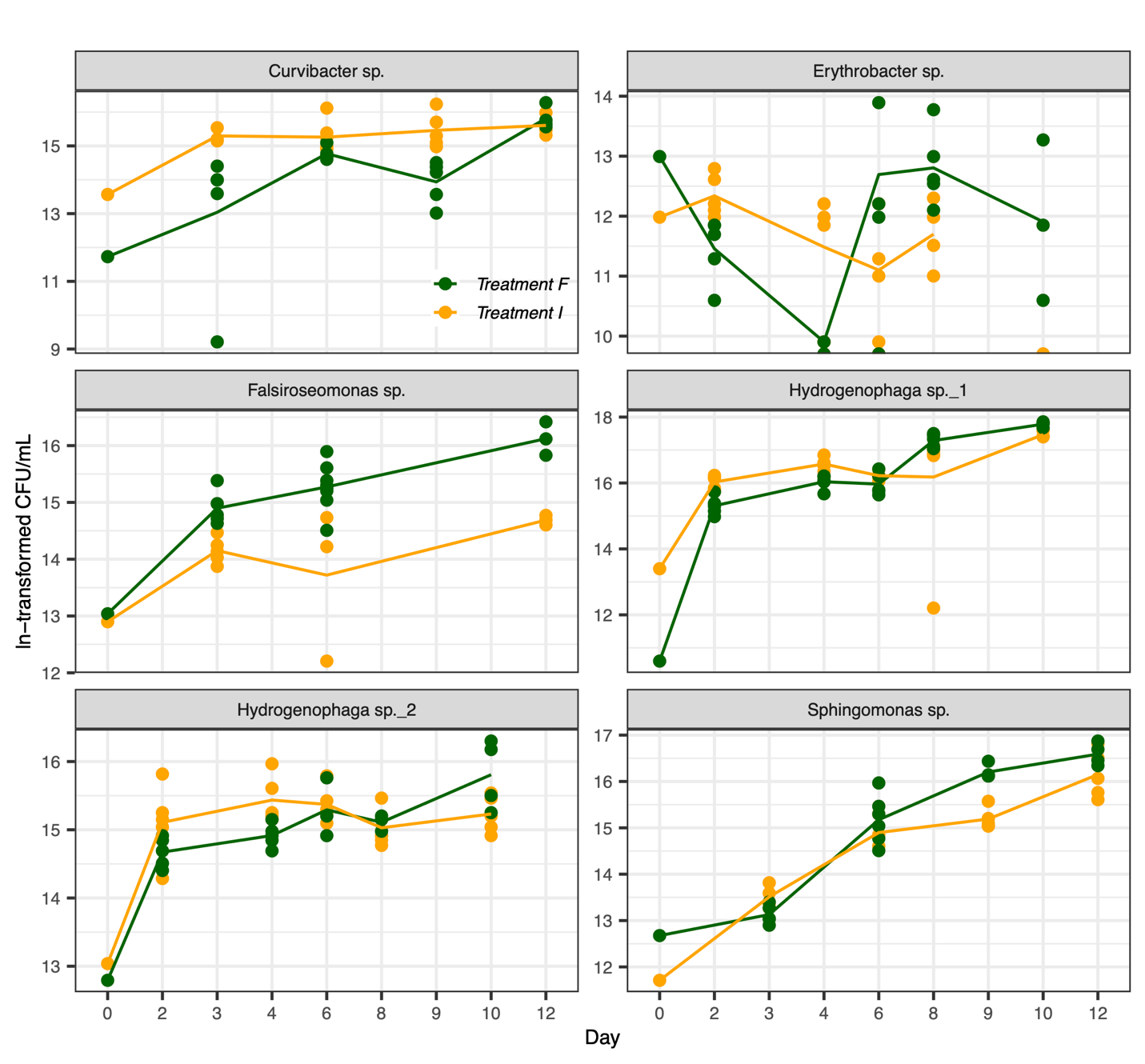
**

**Figure S1.** Growth dynamics of six selected bacteria isolated from the *C. sorokiniana* microbiome. Bacteria were grown with Treatment I and Treatment F in 1-mL culture media. Green and orange points indicate bacterial density based on plate colony forming unit (CFU) under Treatment I and F. Growth curve lines indicate the mean of replicates.

**
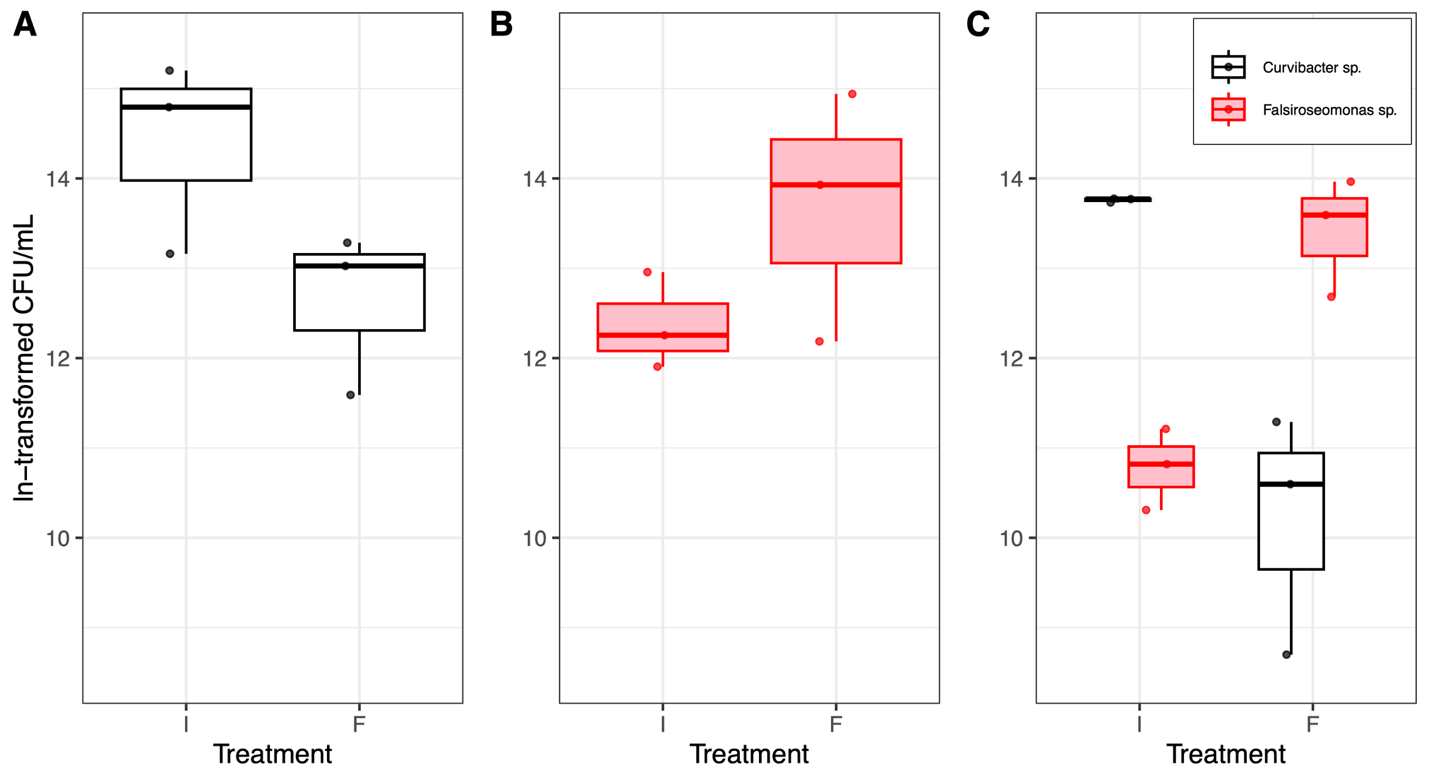
**

**Figure S2.** Bacterial density at the time of collection for transcriptomic analysis when (A) *Curvibacter sp.* was grown alone, (B) *Falsiroseomonas sp.* was grown alone, and (C) a mixture of both bacteria was grown. Treatment I and Treatment F were collected at Day 5 and Day 12, respectively. The height of the box represents the middle 50% of the data, the whiskers extend to 1.5 times the interquartile range from the first or third quartile, the middle line in each box is the median.

**
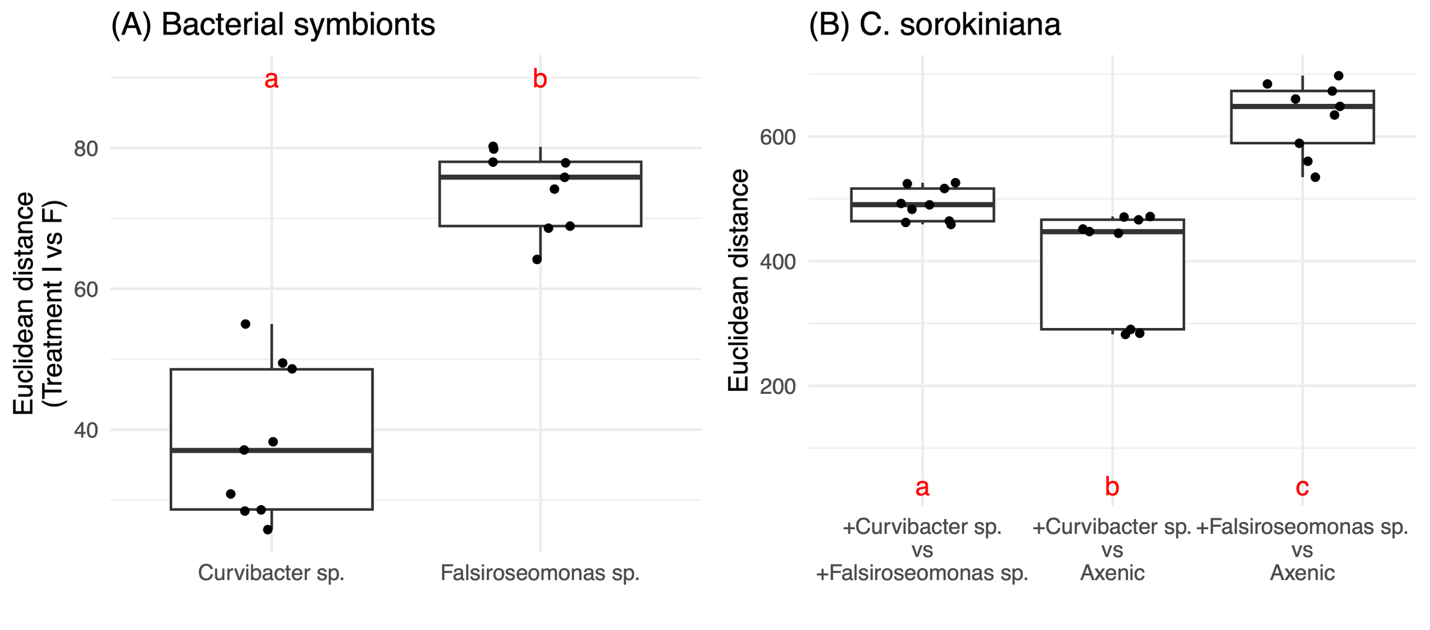
**

**Figure S3.** The Euclidean distance between the variance stabilizing transformed gene/transcript count tables of (A) bacteria between Treatment I and F and (B) *C. sorokiniana* when co-cultured with *Curvibacter* sp. or *Falsiroseomonas* sp*.* and axenic condition. Each point indicates the pairwise comparison among three replicates, resulting in 9 Euclidean distance measures for each comparison. The red letters above each boxplot separate groups with significantly different Euclidean distance between comparisons (Kruskal-Wallis test followed by pairwise t-tests with a Bonferroni, multiple testing correction method; adjusted p-value ⩽ 0.05). The height of the box represents the middle 50% of the data, the whiskers extend to 1.5 times the interquartile range from the first or third quartile, the middle line in each box is the median.

**
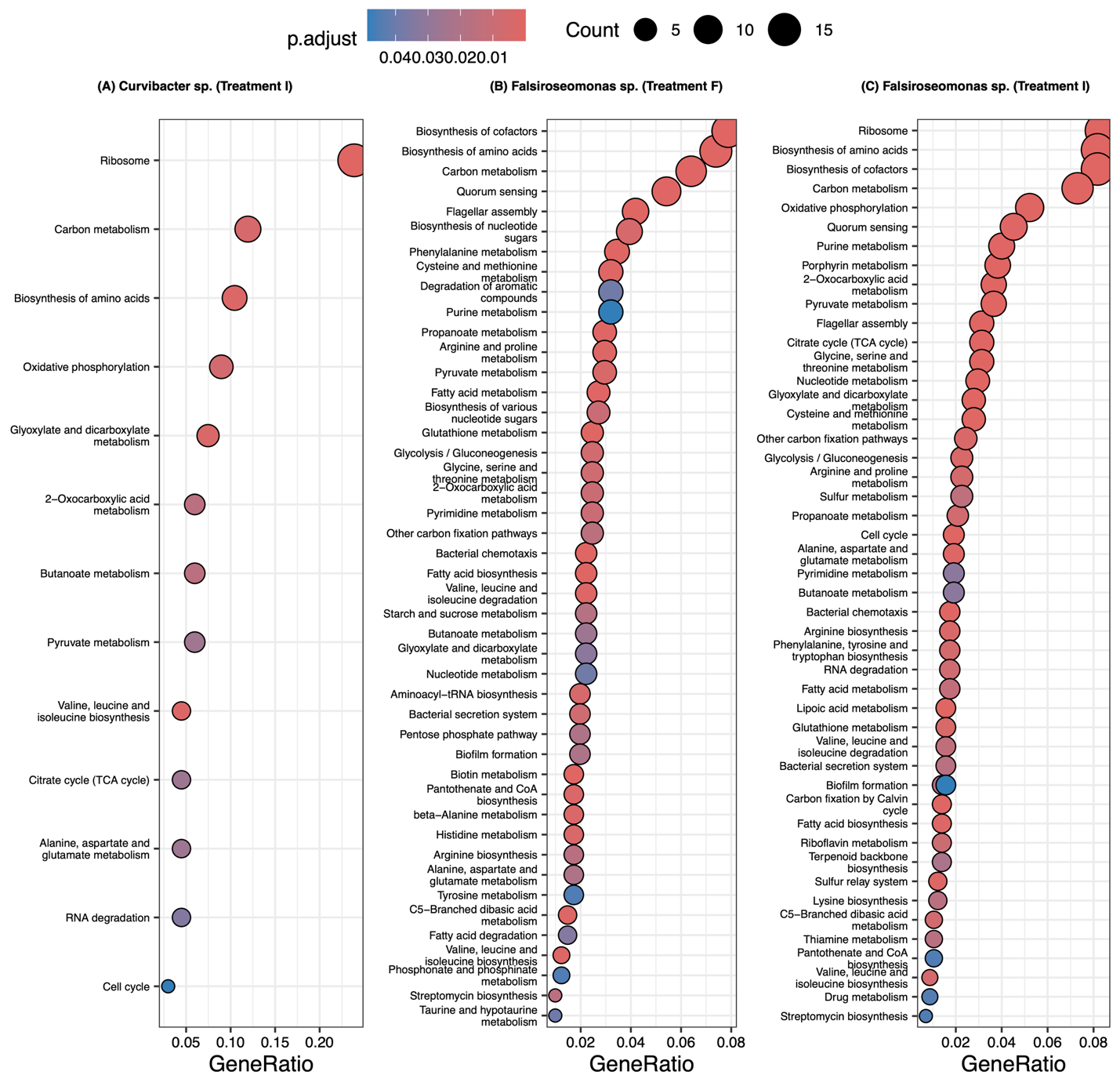
**

**Figure** **S4.** Enrichment analysis on KEGG pathway-annotated DE genes that are more expressed in (A) *Curvibacter* sp. under Treatment I and (B) in *Falsiroseomonas* sp. under Treatment F and (C) Treatment I. GeneRatio were calculated by dividing the number of DE gene assigned to each pathway to the total number of KEGG annotated DE genes in that treatment. The point size indicates the number of DE genes assigned to each pathway. The color of points indicates the adjusted p-value to determine the observed number of DE genes in a given pathway significantly higher than expected by chance (adjusted-p ⩽0.05).

**
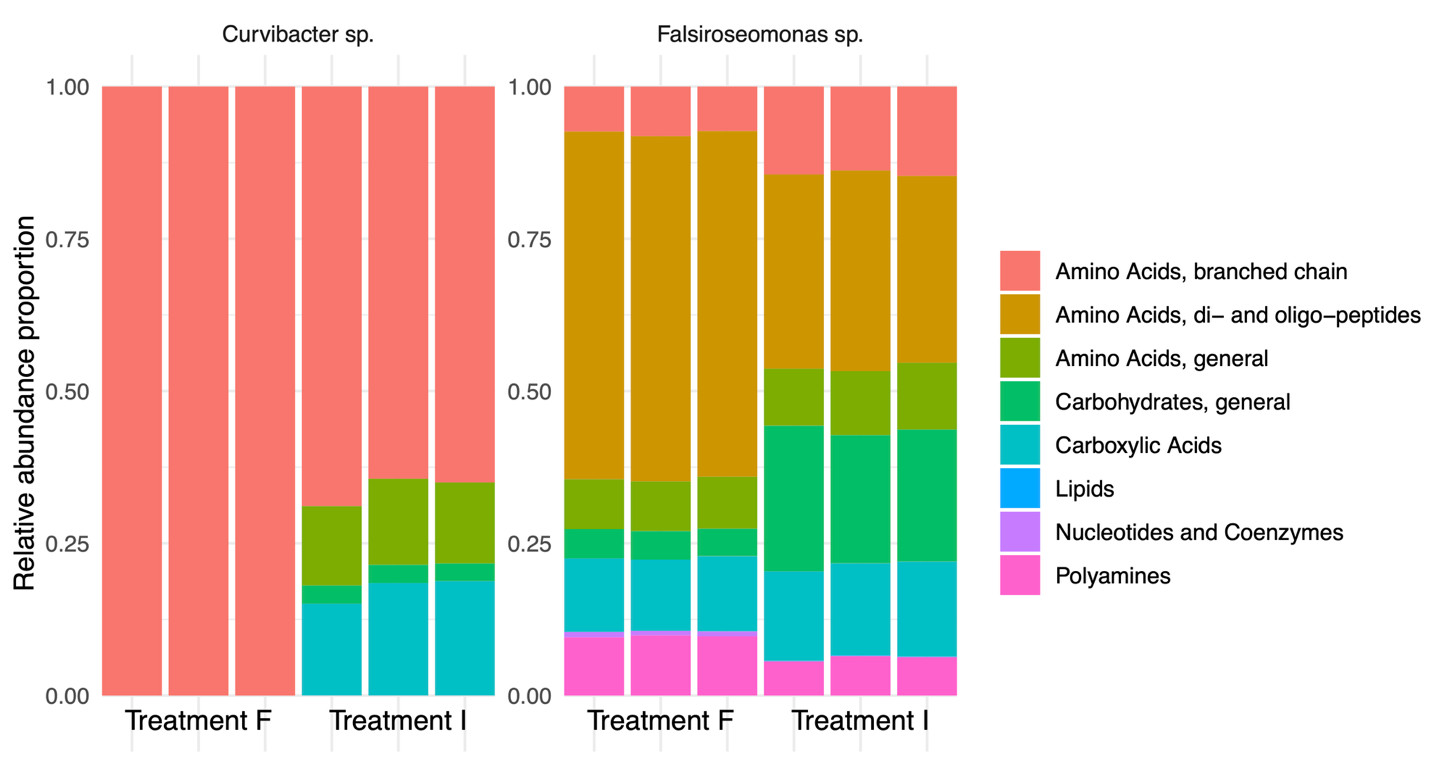
Figure S5.** Proportion of DOM-related transporter DE gene relative abundance. The relative abundance proportion was calculated by dividing the relative abundance by the sum of all relative abundances.

**
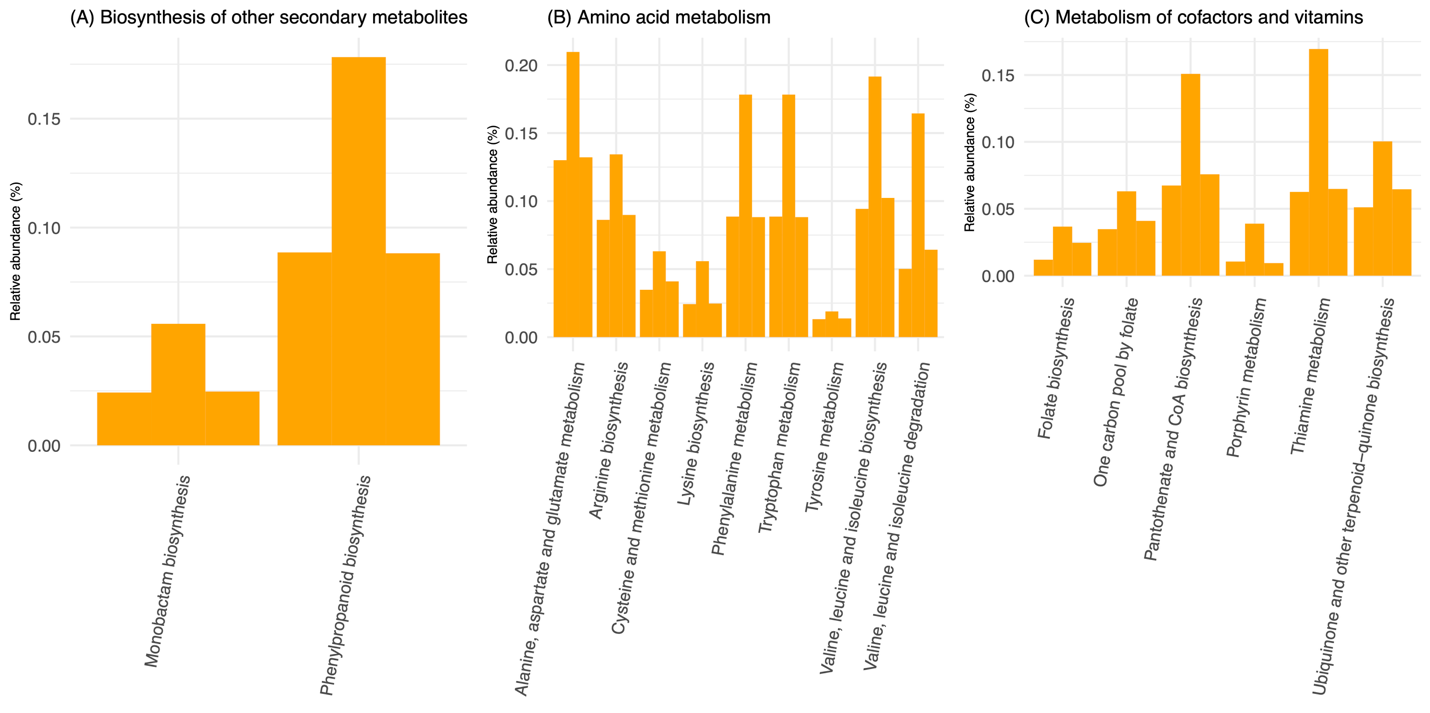
Figure S6.** The relative abundance of KEGG pathway-annotated differentially expressed (DE) genes in *Curvibacter* sp. across three KEGG categories: (A) Biosynthesis of secondary metabolites, (B) Amino acid metabolism, and (C) Metabolism of cofactors and vitamins. Each colored bar represents a replicate for the indicated treatment (three replicates per treatment). Yellow bars representing the relative abundance of DE genes with higher relative abundance under Treatment I.

**
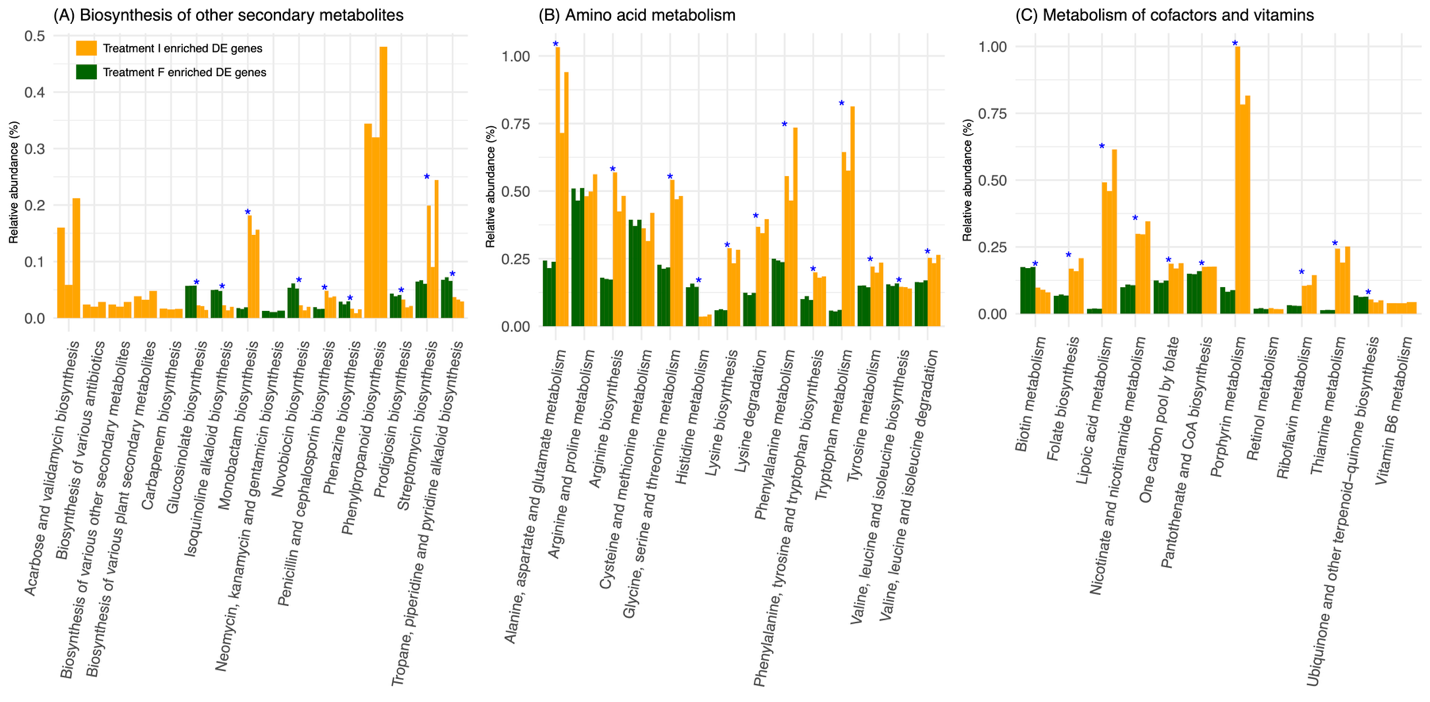
Figure S7.** The relative abundance of KEGG pathway-annotated differentially expressed (DE) genes in *Falsiroseomonas* sp. across three KEGG categories: (A) Biosynthesis of secondary metabolites, (B) Amino acid metabolism, and (C) Metabolism of cofactors and vitamins. Each colored bar represents a replicate for the indicated treatment (three replicates per treatment). Green and yellow bars represent the relative abundance of DE genes with higher relative abundance under Treatment F and I, respectively. Blue asterisks denote significant differences in relative abundance between treatments based on a Wilcoxon test with adjusted p-values ≤ 0.1.

**
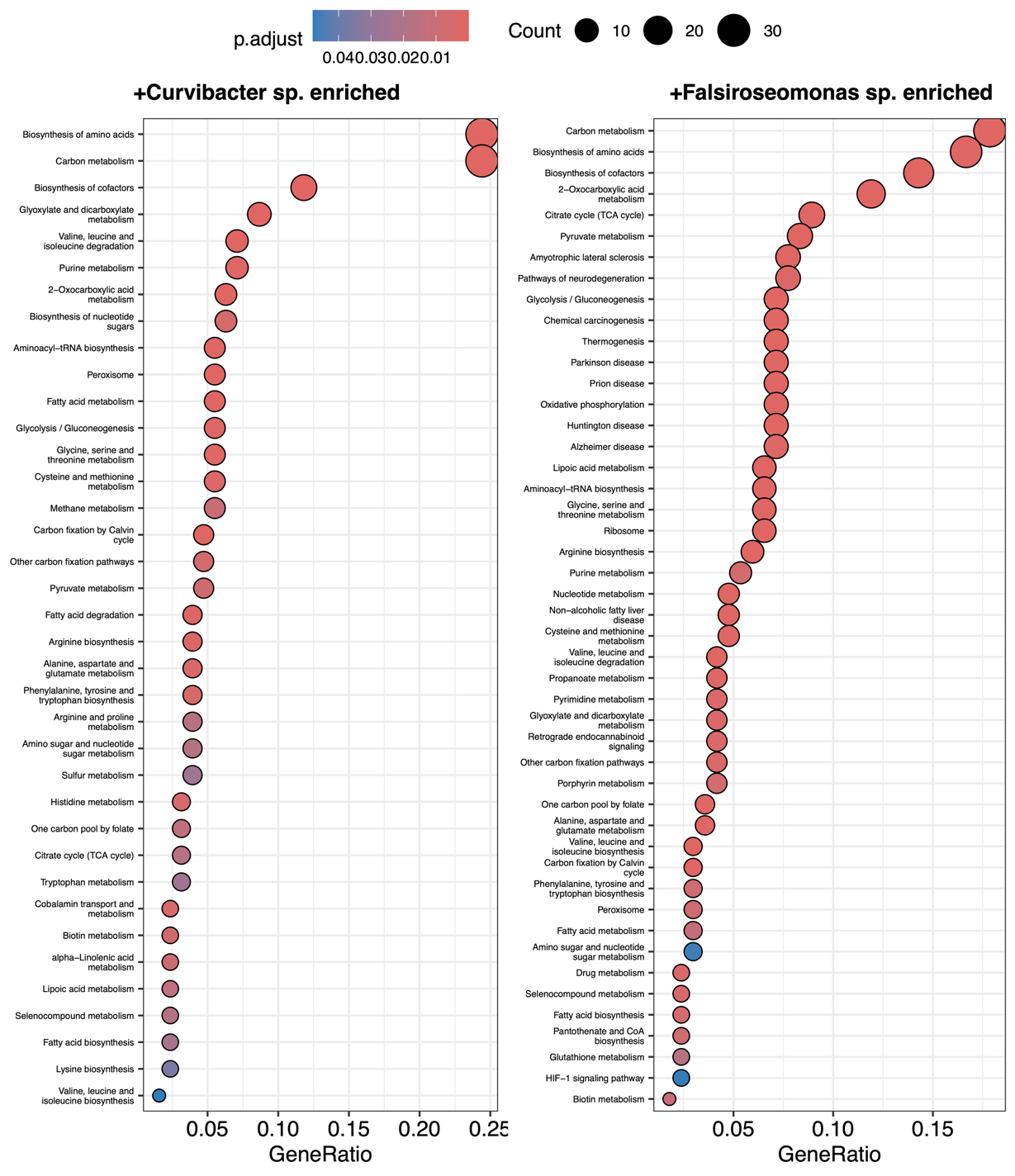
**

**Figure S8.** Enrichment analysis on KEGG pathway-annotated DE genes in *C. sorokiniana.*

GeneRatio were calculated by dividing the number of DE genes assigned to each pathway to the total number of KEGG annotated DE genes in that treatment. The point size indicates the number of DE genes assigned to each pathway. The color of points indicates the adjusted p-value to determine the observed number of DE genes in a given pathway significantly higher than expected by chance (adjusted-p ⩽0.05).

**
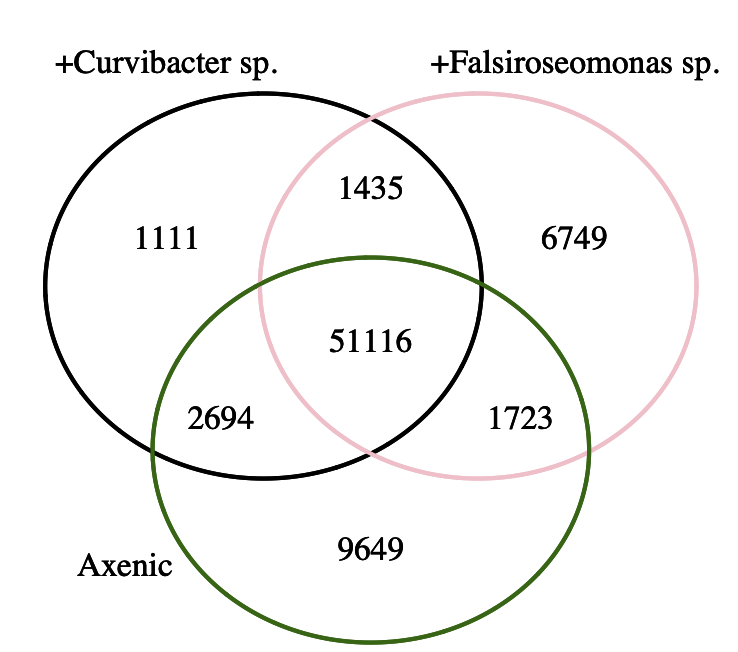
**

**Figure S9.** The number of identified transcripts in *C. sorokiniana* among axenic status and with the presence of either symbiont. The numbers in the green, black and pink circles show the number of identified transcripts when axenic, co-cultured with *Curvibacter* sp., and *Falsirosomonas* sp., respectively.
